# A Novel Method for Mendelian Randomization Analyses With Pleiotropy and Linkage Disequilibrium in Genetic Variants From Individual Data

**DOI:** 10.3389/fgene.2021.634394

**Published:** 2021-07-12

**Authors:** Yuquan Wang, Tingting Li, Liwan Fu, Siqian Yang, Yue-Qing Hu

**Affiliations:** ^1^State Key Laboratory of Genetic Engineering, Human Phenome Institute, Institute of Biostatistics, School of Life Sciences, Fudan University, Shanghai, China; ^2^Shanghai Center for Mathematical Sciences, Fudan University, Shanghai, China

**Keywords:** causal effect, individual data, linkage disequilibrium, Mendelian randomization, mixed-effects model, pleiotropy

## Abstract

Mendelian randomization makes use of genetic variants as instrumental variables to eliminate the influence induced by unknown confounders on causal estimation in epidemiology studies. However, with the soaring genetic variants identified in genome-wide association studies, the pleiotropy, and linkage disequilibrium in genetic variants are unavoidable and may produce severe bias in causal inference. In this study, by modeling the pleiotropic effect as a normally distributed random effect, we propose a novel mixed-effects regression model-based method PLDMR, pleiotropy and linkage disequilibrium adaptive Mendelian randomization, which takes linkage disequilibrium into account and also corrects for the pleiotropic effect in causal effect estimation and statistical inference. We conduct voluminous simulation studies to evaluate the performance of the proposed and existing methods. Simulation results illustrate the validity and advantage of the novel method, especially in the case of linkage disequilibrium and directional pleiotropic effects, compared with other methods. In addition, by applying this novel method to the data on Atherosclerosis Risk in Communications Study, we conclude that body mass index has a significant causal effect on and thus might be a potential risk factor of systolic blood pressure. The novel method is implemented in R and the corresponding R code is provided for free download.

## 1. Introduction

Conventional epidemiology has made enormous contributions to identifying certain significant exposures associated with common diseases, like fine particle air pollution was found to increase the risk of lung cancer mortality (Knowler et al., 2002; Pope et al., 2002). However, some epidemiological findings have later been revealed to be misleading by randomized controlled trials (RCTs) (Smith and Ebrahim, 2005). Furthermore, even if RCTs can correct the bias, despite the high cost of RCTs, the randomization of some potential confounders like nutrition and physical activity may be unfeasible (Smith and Ebrahim, 2003), thus some statistical methods were developed and employed to infer the causal relationship of interested exposures and diseases in epidemiology studies.

Mendelian randomization (MR) applies the method of instrumental variables (IVs) to estimate the causal effect of a non-genetic exposure on a disease outcome (Lawlor et al., 2008). MR exceeds conventional observational epidemiology in many aspects. Just as the role that IVs play in econometrics, setting genetic variants, e.g., single-nucleotide polymorphisms (SNPs), as instrumental variables, MR is capable of excluding the unknown confounders which often interfere with the conventional epidemiological analyses. What is more, not like RCTs spending large amounts of time and money in designing experiments and measuring physiological indexes, MR is practical and economical in the sense of using statistical methods. Methodological studies on MR in recent years have facilitated the reuse of results from genome-wide association studies (GWASs) (Burgess et al., 2013; Bowden et al., 2015, 2016). The GWAS is able to detect association between genetic variants and traits (Visscher et al., 2017). Immense results of GWASs are available through various online databases, such as Gene ATLAS and GWAS Catalog (Buniello et al., 2018; Canela-Xandri et al., 2018), from where we can get summary statistics like effects of SNPs on exposures and outcomes. To discover the causal relationships between exposure-outcome pairs, these statistics are necessary for MR methods. There are also some methods developed to infer causal relationships in individual-level data (Kang et al., 2016; Windmeijer et al., 2019), in addition to the general two-sample MR methods, which can be easily conducted and only require one-sample individual-level data.

Selecting a genetic variant as an IV, we must follow several critical assumptions (Angrist et al., 1996), among which the exclusion restriction assumption implies any effect of an IV on the outcome must be via an effect of the IV on the exposure (i.e., no pleiotropy; Angrist et al., 1996). However, it is possible that pleiotropy occurs in MR studies when taking multiple genetic variants as IVs, as numerous conclusions from GWASs have suggested (Soranzo et al., 2009; Lauc et al., 2013; Hu et al., 2018; Parker et al., 2019; Watanabe et al., 2019). To correct the bias in causal effect estimation produced by the latent pleiotropy of IVs, MR-Egger was proposed and widely employed in MR analyses, which viewed individual IV estimates as separate study results in meta-analysis and applied Egger's regression for interpreting pleiotropy in causal inference (Bowden et al., 2015; Yavorska and Burgess, 2017; Zhan and Fang, 2019). The latest version of the package MendelianRandomization (Yavorska and Burgess, 2017) allows MR-Egger to adjust for the bias brought by the linkage disequilibrium (LD) between genetic variants. However, MR-Egger (Bowden et al., 2015) only considers correcting the average pleiotropic effect, ignoring the potential variance of pleiotropic effects for invalid IVs, which may also influence causal inference. Thus, whether MR-Egger is able to handle LD and random pleiotropic effects simultaneously needs to be verified. LDA MR-Egger (Barfield et al., 2018) improves the performances of MR-Egger when LD exists between genetic variants but still has problems when the variance of pleiotropic effect is considerable. Other two-sample MR methods such as MR-LDP (Cheng et al., 2020) and RAPS (Zhao et al., 2020) are unable to correct the directional pleiotropic effect.

In this paper, we first introduce the mixed-effects regression model inherited from MR-Egger (Bowden et al., 2015) and briefly review MR-Egger method. Then we propose our novel method, pleiotropy and linkage disequilibrium adaptive Mendelian randomization (PLDMR), which models and corrects both the mean and variance of pleiotropic effects, as well as LD between genetic variants in causal effect estimation and statistical testing. We also derive two approximations of PLDMR, i.e., LDMR when the variance of pleiotropic effect is about zero and PLDMR_a_ when the sample size is sufficiently large. We further compare the statistical properties of PLDMR against MR-Egger as well as several two-sample summary-level data methods developed in recent years, such as MR-LDP (Cheng et al., 2020), RAPS (Zhao et al., 2020), and LDA MR-Egger (Barfield et al., 2018), in terms of estimation and statistical testing in various simulation scenarios. Furthermore, we apply PLDMR, LDMR, and PLDMR_a_ to the data of Atherosclerosis Risk in Communications Study (ARIC) and identify the significant causal effect of body mass index (BMI) on systolic blood pressure (SBP). We conclude that incorporating the variance of the pleiotropic effects and LD into MR analyses can efficiently estimate the causal effect and make more credible causal inference.

## 2. Materials and Methods

### 2.1. Mendelian Randomization and Regression Models

Let us first recall the regression models used in MR-Egger (Bowden et al., 2015). For *n* individuals, let the matrix ***G*** = (*G*_*ij*_)_*n*×*m*_ denote their centralized measurement of the *m* genetic variants, where *G*_*ij*_ is the genotype of individual *i* at the *j*th variant, 1 ≤ *i* ≤ *n*, 1 ≤ *j* ≤ *m*. $X={\left(X_{1},X_{2},\ldots ,X_{n}\right)}^{T}$ and $Y={\left(Y_{1},Y_{2},\ldots ,Y_{n}\right)}^{T}$ are centralized measurements of the exposure and outcome of the *n* individuals, respectively. The exposure ***X*** is the linear combination of *m* genotypes and an error term $\varepsilon_{X}={\left(\varepsilon_{X_{1}},\varepsilon_{X_{2}},\ldots ,\varepsilon_{X_{n}}\right)}^{T}$, and the outcome ***Y*** is the linear combination of *m* genotypes, the exposure and an error term $\varepsilon_{Y}={\left(\varepsilon_{Y_{1}},\varepsilon_{Y_{2}},\ldots ,\varepsilon_{Y_{n}}\right)}^{T}$. To simplify the model, we reflect the influence of unknown confounders on ***X*** and ***Y*** in the correlatedness of the error terms **ε**_***X***_ and **ε**_***Y***_. The causal effect of the exposure on the outcome is β in the model, which is of interest. The coefficients $\gamma ={\left(\gamma_{1},\gamma_{2},\ldots ,\gamma_{m}\right)}^{T}$ represent the effect of *m* genetic variants on the exposure, and $\alpha ={\left(\alpha_{1},\alpha_{2},\ldots ,\alpha_{m}\right)}^{T}$ is the pleiotropic effect of *m* genetic variants on the outcome. Specifically,

$$\begin{array}{c} X=G\gamma +\varepsilon_{X},\\ Y=G\alpha +X\beta +\varepsilon_{Y},\\ \left(\begin{array}{c} \varepsilon_{X}\\ \varepsilon_{Y} \end{array}\right)\sim N\left(\left(\begin{array}{c} 0\\ 0 \end{array}\right),\left(\begin{array}{cc} \sigma_{X}^{2} & \rho \sigma_{X}\sigma_{Y}\\ \rho \sigma_{X}\sigma_{Y} & \sigma_{Y}^{2} \end{array}\right)⊗I_{n}\right), \end{array}$$

where σ_*X*_ ∈ (0, ∞), σ_*Y*_ ∈ (0, ∞), ρ ∈ (−1, 1), $\alpha \sim N\left(\mu_{\alpha }1,\sigma_{\alpha }^{2}I_{m}\right)$ is the random pleiotropic effect independent of ***G***, **ε**_***X***_, and **ε**_***Y***_ (Zhao et al., 2020), ***I*** is the identity matrix, ⊗ is the Kronecker product, and **1** is all 1's vector of length *m*. To take genetic variants as valid IVs in the conventional MR studies, the following assumptions should be satisfied (Angrist et al., 1996): (i) The genetic variants are randomly assigned, thus independent of unknown confounders; (ii) The genetic variants should be associated with the exposure; (iii) Any effect of the genetic variants on the outcome must be via an effect of that on the exposure. Equivalently speaking, (i) assumes ***G*** is independent of **ε**_***X***_ and **ε**_***Y***_; (ii) requires γ_*j*_ ≠ 0 for each genetic variant *j*, which can be met by selecting genetic variants with methods like linear regression; (iii) implies no pleiotropic effect, i.e., **α** = **0**. Our aim is to estimate the causal effect β and then make the statistical inference on it. To this end, we employ the mixed-effects model as described above to relax the requirement in (iii).

### 2.2. Revisit Egger Regression and MR-Egger

Let ${\tilde{\Gamma }}_{j}$ and ${\tilde{\gamma }}_{j}$ denote the coefficient estimates of the simple linear regression of the outcome ***Y*** and the exposure ***X*** on the genotype $G_{.j}={\left(G_{1j},G_{2j},\ldots ,G_{nj}\right)}^{T}$ at variant *j*, respectively, and $SE\left({\tilde{\Gamma }}_{j}\right)$ denote the standard error of ${\tilde{\Gamma }}_{j},1\leq j\leq m$. An adaption of Egger regression was proposed (Bowden et al., 2015) as follows to estimate the causal effect,

$$\begin{array}{r} \tilde{\Gamma }=\beta_{0E}1+\beta_{E}\tilde{\gamma }+e_{\tilde{\Gamma }},\;e_{\tilde{\Gamma }}\\ \sim N(0,\sigma^{2}\text{diag}\left(SE^{2}\left({\tilde{\Gamma }}_{1}\right),SE^{2}\left({\tilde{\Gamma }}_{2}\right),\ldots ,SE^{2}\left({\tilde{\Gamma }}_{m}\right)\right), \end{array}$$

where $\tilde{\Gamma }={\left({\tilde{\Gamma }}_{1},{\tilde{\Gamma }}_{2},\ldots ,{\tilde{\Gamma }}_{m}\right)}^{T}$, $\tilde{\gamma }={\left({\tilde{\gamma }}_{1},{\tilde{\gamma }}_{2},\ldots ,{\tilde{\gamma }}_{m}\right)}^{T}$.

Imposing the constraint of β_0*E*_ = 0 on the above regression model yields the inverse-variance weighted (IVW) estimate of the causal effect (Burgess et al., 2013), which is also commonly used in the meta-analysis. Notice that both MR-Egger and IVW are applicable to the summary data that are accessible in most GWASs.

### 2.3. PLDMR Adjusted for Pleiotropy and Linkage Disequilibrium

With the rapidly increasing number of genetic variants involved in Mendelian randomization studies, it is necessary to take the correlation among variants into account in estimating the causal effect of exposure on the outcome. Instead of the marginal regression of exposure/outcome on the genotype, multiple linear regression of ***Y*** on ***G***, and ***X*** on ***G*** are employed to derive the coefficient estimates $\hat{\Gamma }$ and $\hat{\gamma }$, respectively. To be precise, $\hat{\Gamma }={\left(G^{T}G\right)}^{-1}G^{T}Y$ and $\hat{\gamma }={\left(G^{T}G\right)}^{-1}G^{T}X$. Further, we have

$$\begin{array}{l} \hat{\Gamma }={\left(G^{T}G\right)}^{-1}G^{T}\left(G\alpha +X\beta +\varepsilon_{Y}\right)\\ \;=\alpha +\beta \hat{\gamma }+{\left(G^{T}G\right)}^{-1}G^{T}\varepsilon_{Y}\\ \;=\mu_{\alpha }1+\beta \hat{\gamma }+\left(\alpha -\mu_{\alpha }1\right)+{\left(G^{T}G\right)}^{-1}G^{T}\varepsilon_{Y}. \end{array}$$

Based on the independence of **α** and **ε**_***Y***_ and also their normality, we have the following regression model

$$\begin{array}{l} \hat{\Gamma }=\mu_{\alpha }1+\beta \hat{\gamma }+\varepsilon_{\hat{\Gamma }},\;\varepsilon_{\hat{\Gamma }}\sim N\left(0,W^{-1}\right), \end{array}$$

where $W={\left[\sigma_{\alpha }^{2}I_{m}+\sigma_{Y}^{2}{\left(G^{T}G\right)}^{-1}\right]}^{-1}$. The corresponding likelihood function is

$$\begin{array}{l} L\left(\mu_{\alpha },\beta ,\sigma_{\alpha }^{2},\sigma_{Y}^{2};\hat{\Gamma },\hat{\gamma }\right)\\ ={\left(2\pi \right)}^{-\frac{m}{2}}|W{|}^{\frac{1}{2}}\text{exp}\left[-\frac{1}{2}{\left(\hat{\Gamma }-\mu_{\alpha }1-\beta \hat{\gamma }\right)}^{T}W\left(\hat{\Gamma }-\mu_{\alpha }1-\beta \hat{\gamma }\right)\right]. \end{array}$$

Notice that both unknown parameters $\sigma_{\alpha }^{2}$ and $\sigma_{Y}^{2}$ are involved in ***W***, which renders difficulty in the calculation of the maximum likelihood estimates (MLEs). For the positive definite matrix (***G***^*T*^***G***)^−1^, there exists an *m* × *m* orthogonal matrix ***Q*** and an *m* × *m* diagonal matrix **Λ** such that (***G***^*T*^***G***)^−1^ = ***Q*****Λ*****Q***^*T*^. Let $r^{2}=\sigma_{\alpha }^{2}/\sigma_{Y}^{2}$, we then express ***W***^−1^ as $\sigma_{Y}^{2}\left(r^{2}I_{m}+{Q\Lambda Q}^{T}\right)$ and further diagonalize ***Q***^*T*^***W***^−1^***Q*** as $\sigma_{Y}^{2}\left(r^{2}I_{m}+\Lambda \right)$. So the likelihood function is transformed to

$$\begin{array}{l} L\left(\mu_{\alpha },\beta ,r^{2},\sigma_{Y}^{2};\hat{\Gamma },\hat{\gamma }\right)={\left(2\pi \sigma_{Y}^{2}\right)}^{-\frac{m}{2}}|r^{2}I_{m}+\Lambda {|}^{-\frac{1}{2}}\cdot \\ \text{exp }[-\frac{1}{2\sigma_{Y}^{2}}{\left(Q^{T}\hat{\Gamma }-\mu_{\alpha }Q^{T}1-\beta Q^{T}\hat{\gamma }\right)}^{T}{\left(r^{2}I_{m}+\Lambda \right)}^{-1}(Q^{T}\hat{\Gamma }\\ -\mu_{\alpha }Q^{T}1-\beta Q^{T}\hat{\gamma })]. \end{array}$$

We call the R package BB (Varadhan and Gilbert, 2009) implementing the spectral projected gradient algorithm (Varadhan and Roland, 2008) to get the MLEs ${\hat{\mu }}_{\alpha },\hat{\beta }$, and ${\hat{r}}^{2}$. As

$$\begin{array}{r} \hat{\beta }=\frac{{\hat{\gamma }}^{T}W^{\frac{1}{2}}\left(I_{m}-P_{W^{\frac{1}{2}}1}\right)W^{\frac{1}{2}}\hat{\Gamma }}{{\hat{\gamma }}^{T}W^{\frac{1}{2}}\left(I_{m}-P_{W^{\frac{1}{2}}1}\right)W^{\frac{1}{2}}\hat{\gamma }}\\ \sim N\left(\beta ,\frac{1}{{\hat{\gamma }}^{T}W^{\frac{1}{2}}\left(I_{m}-P_{W^{\frac{1}{2}}1}\right)W^{\frac{1}{2}}\hat{\gamma }}\right), \end{array}$$

where $P_{W^{\frac{1}{2}}1}=\frac{W^{\frac{1}{2}}11^{T}W^{\frac{1}{2}}}{1^{T}W1}$ is the orthogonal projection onto $W^{\frac{1}{2}}1$. The plug-in method is invoked to get $\hat{Var}\left(\hat{\beta }\right)$, the estimate of the variance of $\hat{\beta }$. Based on these estimates, we have approximately

$$\begin{array}{l} \frac{\hat{\beta }-\beta }{\sqrt{\hat{Var}\left(\hat{\beta }\right)}}\sim t\left(m-2\right), \end{array}$$

which can easily yield the confidence interval of the causal effect β or the *p*-value in testing the statistical hypothesis *H*_0_ : β = β_0_, where *t*(*m* − 2) represents the *t*-distribution with *m* − 2 degrees of freedom. We use PLDMR for the statistical inference of the causal effect in the presence of pleiotropy and multiple SNPs in LD in Mendelian randomization analyses.

Considering the sample size *n* is usually an order of tens of thousands, we have ***G***^*T*^***G*** = *O*(*n*) and further $W\approx \sigma_{\alpha }^{-2}I_{m}$. As an approximation in the situation of big *n*, the estimate of the causal effect β and its variance are easily derived from classical simple linear regression. We denote this approximation as PLDMR_a_. The accuracy of this approximation is illustrated in the simulation study for varied sample sizes from several hundreds to several tens of thousands and varied $\sigma_{\alpha }^{2}$.

Another special case of our interest is $\sigma_{\alpha }^{2}=0$, i.e., **α** = μ_α_ **1**, or $\sigma_{\alpha }^{2}\approx 0$. We have $W\approx \sigma_{Y}^{-2}\left(G^{T}G\right)$ and then the estimates of the causal effect and its variance can be derived approximately from the following simple linear regression

$$\begin{array}{l} \hat{\Gamma }=\mu_{\alpha }1+\beta \hat{\gamma }+\varepsilon_{\hat{\Gamma }},\varepsilon_{\hat{\Gamma }}\sim N\left(0,\sigma_{Y}^{2}{\left(G^{T}G\right)}^{-1}\right). \end{array}$$

So regressing $\hat{\Gamma }$ on $\hat{\gamma }$ yields

$$\begin{array}{l} {\hat{\beta }}_{LDMR}=\frac{{\hat{\gamma }}^{T}\left(G^{T}G-\frac{G^{T}GJG^{T}G}{1^{T}G^{T}G1}\right)\hat{\Gamma }}{{\hat{\gamma }}^{T}\left(G^{T}G-\frac{G^{T}GJG^{T}G}{1^{T}G^{T}G1}\right)\hat{\gamma }}, \end{array}$$

where ***J*** is all 1's *m* × *m* matrix, and further

$$\begin{array}{l} {\hat{\beta }}_{LDMR}\sim N\left(\beta ,\frac{1}{{\hat{\gamma }}^{T}{\left(G^{T}G\right)}^{\frac{1}{2}}\left(I_{m}-P_{{\left(G^{T}G\right)}^{\frac{1}{2}}1}\right){\left(G^{T}G\right)}^{\frac{1}{2}}\hat{\gamma }}\right). \end{array}$$

Again, we can use this normality to construct the confidence interval of β or test the statistical hypothesis of β when the variance of pleiotropic effect is about zero. We refer to this method as LDMR. In contrast to PLDMR, the estimators of LDMR and PLDMR_a_ have closed forms and thus have no computational burden.

### 2.4. The Design of Simulation Studies

To evaluate the proposed methods, a series of scenarios of different parameter settings are designed to conduct the simulation studies. We explore and compare the estimation results and statistical properties of MR-LDP, RAPS, MR-Egger, and LDA MR-Egger with PLDMR in nine combinations of three patterns of pleiotropy (balanced, negative and positive) and three magnitudes of linkage disequilibrium (no, low, and high). We also vary *n*, the sample size, and $\sigma_{\alpha }^{2}$, the variance of pleiotropic effect, to illustrate whether PLDMR can be approximated by LDMR and PLDMR_a_ in the two situations, i.e., $\sigma_{\alpha }^{2}\approx 0$ and large *n*, respectively. Additionally, we generate genotype data in LD for every individual *i* as the steps listed below:

- Construct a Toeplitz *m* × *m* matrix **Σ**_***g***_, i.e., the (*j*_1_, *j*_2_) cell element is $\rho_{g}^{\left|j_{1}-j_{2}\right|},1\leq j_{1},j_{2}\leq m$;
- Randomly draw $z_{i}={\left(z_{i1},z_{i2},\ldots ,z_{im}\right)}^{T}$ from *MVN*(0, **Σ**_***g***_) and calculate Φ(*z*_*ij*_), where Φ denotes the cumulative distribution function of *N*(0, 1), 1 ≤ *i* ≤ *n*, 1 ≤ *j* ≤ *m*;
- For the given minor allele frequency MAF_*j*_ at the *j*th locus, assign genotype *G*_*ij*_ as the Φ(*z*_*ij*_) quantile of Binomial(2, MAF_*j*_), 1 ≤ *j* ≤ *m*.

The Toeplitz matrix used in (i) is able to weaken the correlation of genotypes at two loci *j*_1_, *j*_2_ with respect to their “relative distance” |*j*_1_ − *j*_2_|. Also, we can control the relative strength of linkage disequilibrium by tuning the magnitude of ρ_*g*_.

## 3. Results

### 3.1. Comparison of Statistical Properties Between PLDMR and Existing Methods

**Figure 1:**
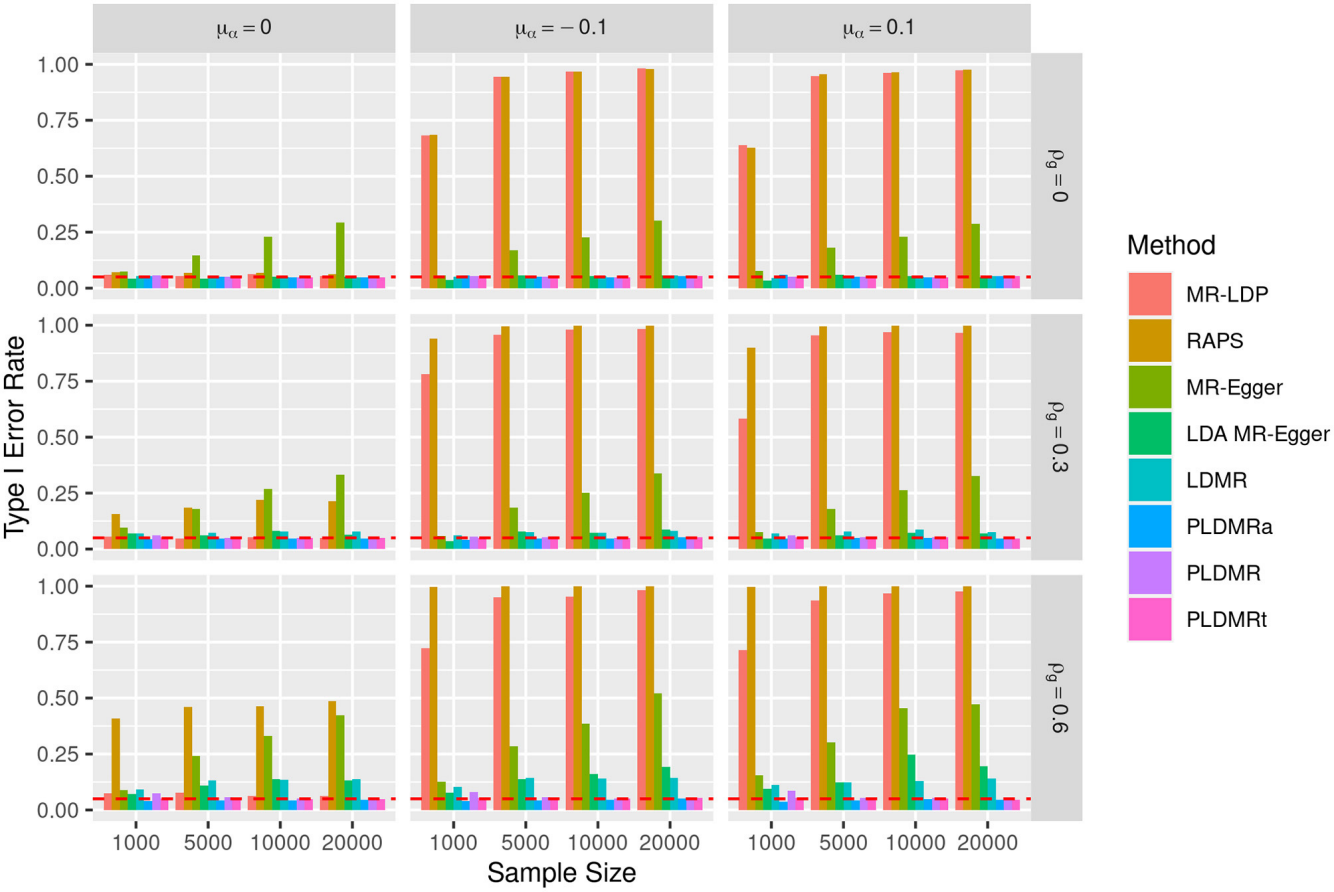
Bar plot of the type I error rates of all methods under the null hypothesis of *H*_0_ : β = 0. Sample size (*n* = 1,000, 5,000, 10,000, 20,000), the number of genetic variants *m* = 25, and σ_α_ = 0.1. μ_α_ = 0, −0.1, 0.1 represents the mean of pleiotropic effect and ρ_*g*_ = 0, 0.3, 0.6 stands for the relative strength of LD between the genetic variants. The red dashed horizontal line indicates the nominal significance of 0.05.

**Figure 2:**
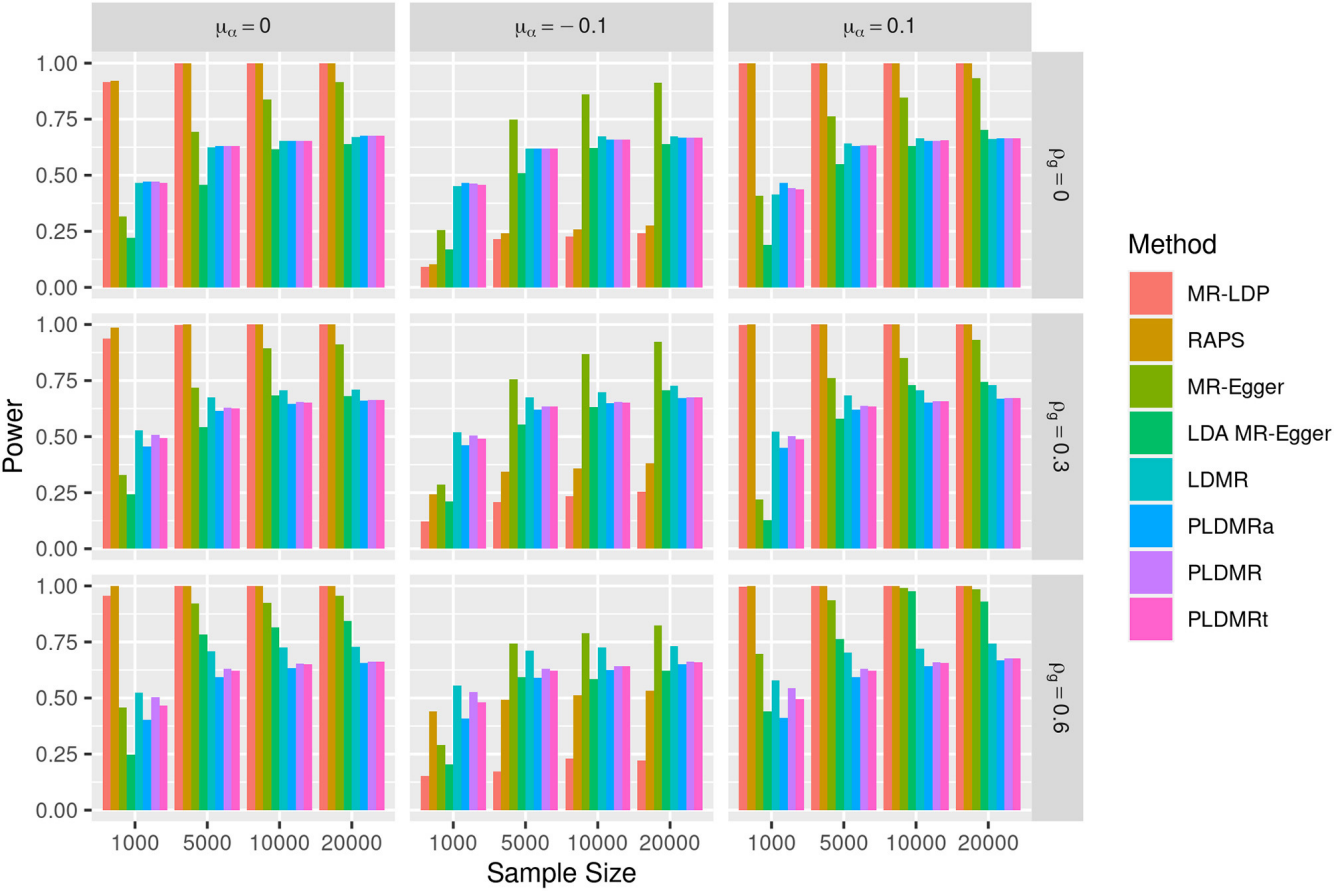
Bar plot of the powers of all methods under the alternative hypothesis of *H*_1_ : β = 0.05. Sample size *n* = 1,000, 5,000, 10,000, 20,000, the number of genetic variants *m* = 25, and σ_α_ = 0.1. μ_α_ = 0, −0.1, 0.1 represents the mean of pleiotropic effect and ρ_*g*_ = 0, 0.3, 0.6 stands for the relative strength of LD between the genetic variants.

All of the methods are implemented using R software (version 3.6.0). To evaluate the proposed methods comprehensively, we choose MAF_*j*_ ~ Uniform([0.2, 0.4]), γ_*j*_ ~ Uniform([0.5, 4]), 1 ≤ *j* ≤ *m*, and fix σ_*X*_ = σ_*Y*_ = 2, ρ = 0.5. We further set ρ_*g*_ = 0, 0.3, and 0.6 to represent no LD, low LD, and high LD; μ_α_ = 0, +0.1, and −0.1 to represent balanced pleiotropy, positive pleiotropy, and negative pleiotropy; σ_α_ = 0.01, 0.1 and 0.2 to represent different strengths of the pleiotropic effect; *n* = 1,000, 5,000, 10,000, and 20,000 to represent a wide range of sample sizes. The nominal significance level is 0.05 and the replications are 10,000 for each scenario. For brevity, results of the simulation study with σ_α_ = 0.1 and *m* = 25 are shown in Figures 1, 2, and the remainders are shown in Supplementary Material.

Four existing two-sample summary-level data methods, i.e., MR-LDP (Cheng et al., 2020), RAPS (Zhao et al., 2020), MR-Egger (Bowden et al., 2015), and LDA MR-Egger (Barfield et al., 2018), are also included in the comparison. Summary level data is obtained by splitting the one-sample individual data into two halves and then conducting simple linear regression in each part. The reference LD correlation matrix needed for these methods is generated from the genotypes of an additional independent 5,000 individuals. We use the R packages MendelianRandomization (version 0.4.2) (Yavorska and Burgess, 2017), MR.LDP (version 1.0), mr.raps (version 0.3.1) to implement the above methods. The code of LDA MR-Egger (Barfield et al., 2018) is downloaded from the github homepage of the author. In addition to PLDMR, LDMR, and PLDMR_a_, we also add PLDMR_t_, which represents the PLDMR method evaluated at the true values of $\sigma_{\alpha }^{2}$ and $\sigma_{Y}^{2}$ instead of the estimated ones in weighted linear regression.

Now let us look at the performances of the eight methods mentioned above in terms of estimation and testing when the true value of β is 0. As is shown in Figure 1, MR-LDP controls type I error rate well in the scenarios of balanced pleiotropy (left panel), but fails in the scenarios of directional pleiotropy (mid and right panels). RAPS fails to control type I error rate when LD or directional pleiotropy exists and only controls the type I error rate in the top-left figure. The type I error rate of MR-Egger method inflates as the sample size increases in each scenario. The type I error rates of LDA MR-Egger and LDMR behave similarly in each scenario, albeit there exists some inflation in the scenarios of high LD (bottom panel). No obvious inflation can be observed from the type I error rates of PLDMR, PLDMR_a_ and PLDMR_t_, although PLDMR_a_ shows some conservativeness in the scenarios of high LD. Supplementary Figure 1 shows the estimation performance of all methods. MR-LDP and RAPS are biased in the scenarios of directional pleiotropy (mid and right panels). MR-Egger and LDA MR-Egger behave similarly in each scenario, as they are both severely biased in the scenarios of directional pleiotropy and high LD (bottom-mid and bottom-right figures). LDMR, PLDMR, and PLDMR_a_ are unbiased in each scenario. However, the standard errors of MR-LDP and RAPS are apparently smaller than those of other methods in the scenarios of balanced pleiotropy (left panel).

Figure 2 depicts the power of detecting the causal effect when β = 0.05. The powers of MR-LDP are higher than LDMR, PLDMR, PLDMR_a_, and PLDMR_t_ in the scenarios of balanced pleiotropy (left panel), but are invalid in the scenarios of directional pleiotropy due to its failure in controlling type I error rates. RAPS is the most powerful method for detecting the causal effect in the scenario of balanced pleiotropy and no LD (top-left figure), but is doubtful in other cases. MR-Egger can control type I error rates only when sample size is small and the correlation between SNPs is low (ρ_*g*_ = 0, 0.3), in which cases its power is lower than LDMR, PLDMR, PLDMR_a_, and PLDMR_t_. LDA MR-Egger performs better than LDMR, PLDMR, PLDMR_a_, and PLDMR_t_ when sample size is large and the correlation between SNPs is low. Supplementary Figure 2 shows the performances of estimations when β = 0.05, which exhibits a similar pattern as when β = 0.

**Figure 3:**
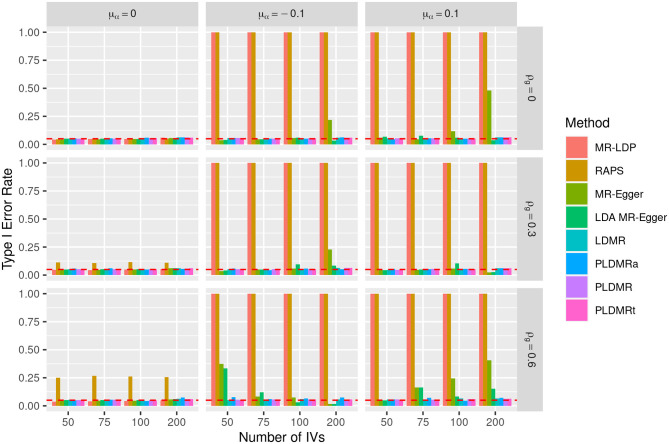
Bar plot of the type I error rates of all methods under the null hypothesis of *H*_0_ : β = 0. Sample size is *n* = 5,000, σ_α_ = 0.01, and the number of genetic variants *m* = 50, 75, 100, 200. μ_α_ = 0, −0.1, 0.1 represents the mean of pleiotropic effect and ρ_*g*_ = 0, 0.3, 0.6 stands for the relative strength of LD between the genetic variants. The red dashed horizontal line indicates the nominal significance of 0.05.

**Figure 4:**
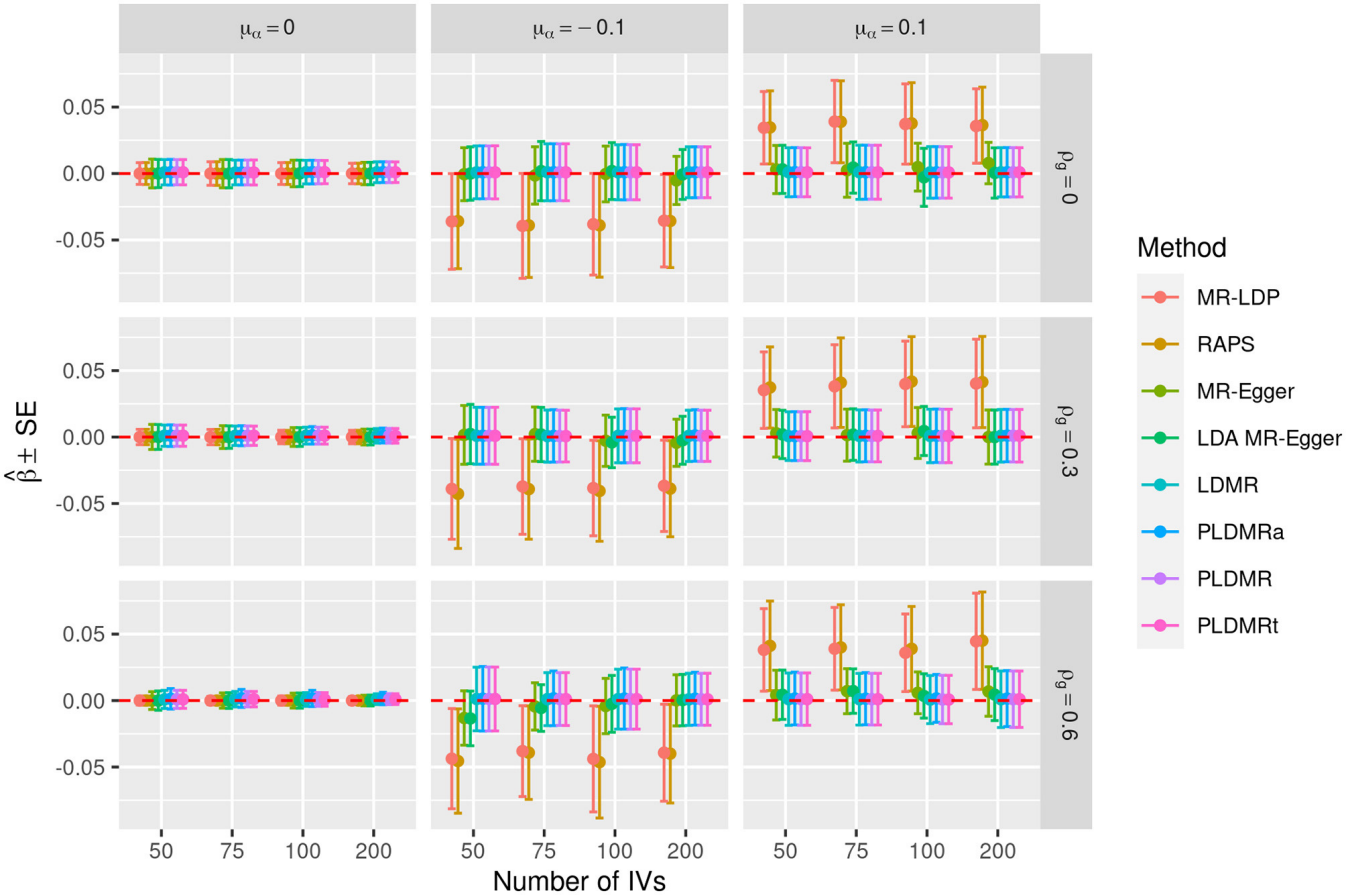
Plot of the performances of all eight estimating methods when β = 0. Sample size is *n* = 5, 000, σ_α_ = 0.01, and the number of genetic variants *m* = 50, 75, 100, 200. μ_α_ = 0, −0.1, 0.1 represents the mean of pleiotropic effect and ρ_*g*_ = 0, 0.3, 0.6 stands for the relative strength of LD between the genetic variants. The solid circles are the mean values of estimators, the upper and lower bars are the means plus and minus one standard error in 10,000 replications. The red dashed line indicates the true value of β.

Figures 3, 4 show the performances of the eight methods using different numbers of IVs. We fix sample size *n* at 5,000 in this simulation and the variance of the pleiotropic effect is σ_α_ = 0.01. MR-LDP still fails to control the type I error rate in the scenarios of directional pleiotropy and RAPS is unable to control type I error rate either when LD exists or directional pleiotropy exists. It can be obviously observed that the type I error rates of MR-Egger inflates when directional pleiotropic effect exists, whereas LDA MR-Egger fails to control type I error rate in the scenarios of directional pleiotropic effect and strong LD. LDMR, PLDMR, PLDMR_a_, and PLDMR_t_ control type I error rates at 0.05 and show no noticeable changes as *m* increases. In Figure 4, the standard errors of all methods decrease with respect to the number of IVs *m*, except for MR-LDP and RAPS in the scenarios of directional pleiotropic effect.

In addition, we compare the type I error rates of all methods under different settings of σ_α_ in Supplementary Figures 3, 5. In Supplementary Figure 3 with σ_α_ = 0.01, MR-Egger can control type I error rate at 0.05 in situations of balanced pleiotropy and no LD but still fails in situations of directional pleiotropy, low and high LD groups. LDMR, PLDMR, and PLDMR_t_ control type I error rates well at around 0.05, whereas PLDMR_a_ obviously is conservative, especially in high LD situations. Except for the conservativeness showed by MR-LDP when sample size is small in the scenarios of balanced pleiotropy, the behaviors of MR-LDP and RAPS are almost the same as those when σ_α_ = 0.1. When σ_α_ = 0.2, the conclusion is similar to that when σ_α_ = 0.1 (Supplementary Figure 5). Furthermore, the powers of all methods when σ_α_ = 0.01 and 0.2 are also shown in Supplementary Figures 7, 9, from where we can conclude that the increasing magnitude of the powers of LDMR, PLDMR, and PLDMR_a_ with respect to sample size under large σ_α_ is much slower than that with smaller σ_α_. The behaviors of estimations of all methods are shown by Supplementary Figures 4, 6, 8, 10.

### 3.2. Briefing and Preprocessing of ARIC Data

Nowadays obesity has become a key issue of global concern (Xu and Lam, 2018). In studying obesity, we usually use BMI to define overweight and obesity. So it is an important factor to use BMI in the relevant research. In order to investigate the causal effect of BMI on SBP and glucose (GLU), we use data on 15,792 individuals from ARIC study. The ARIC study is one of the largest multi-ethnic sampling frame studies in the United States. Nearly 70% of the participants were European Americans, and the rest were African Americans. ARIC includes 909,622 SNPs and more than 450 phenotypes.

Regarding BMI as an exposure and choosing SNPs significantly associated with BMI (*p*-value < 5 × 10^−8^) with reference to GWAS Catalog database (We also choose *p* < 1 × 10^−4^ as another threshold and the corresponding results are shown in Supplementary Table 2), we identify 616 significant SNPs as instrumental variables from ARIC dataset for MR analysis. We only consider individuals of white origin in the following analysis for avoiding the population stratification problem. After model checking, BMI follows normal distribution and it is necessary to log-transform SBP and GLU. We adjust for covariates including sex, age, and age^2^ by regressing BMI, SBP, GLU on these covariates, respectively, and then use the corresponding regression residuals as the adjusted BMI, adjusted SBP and adjusted GLU for the subsequent analysis. After pruning out SNPs with missing value proportion >20% and testing for Hardy-Weinberg equilibrium of the candidate SNPs, multiple linear regression is employed to select genetic variants positively associated with the exposure BMI. Finally, 21 SNPs (see details in Supplementary Table 1) and 6,782 individuals are included in this study after the preliminary processing of data.

### 3.3. Causal Inference of BMI on SBP

**Figure 5:**
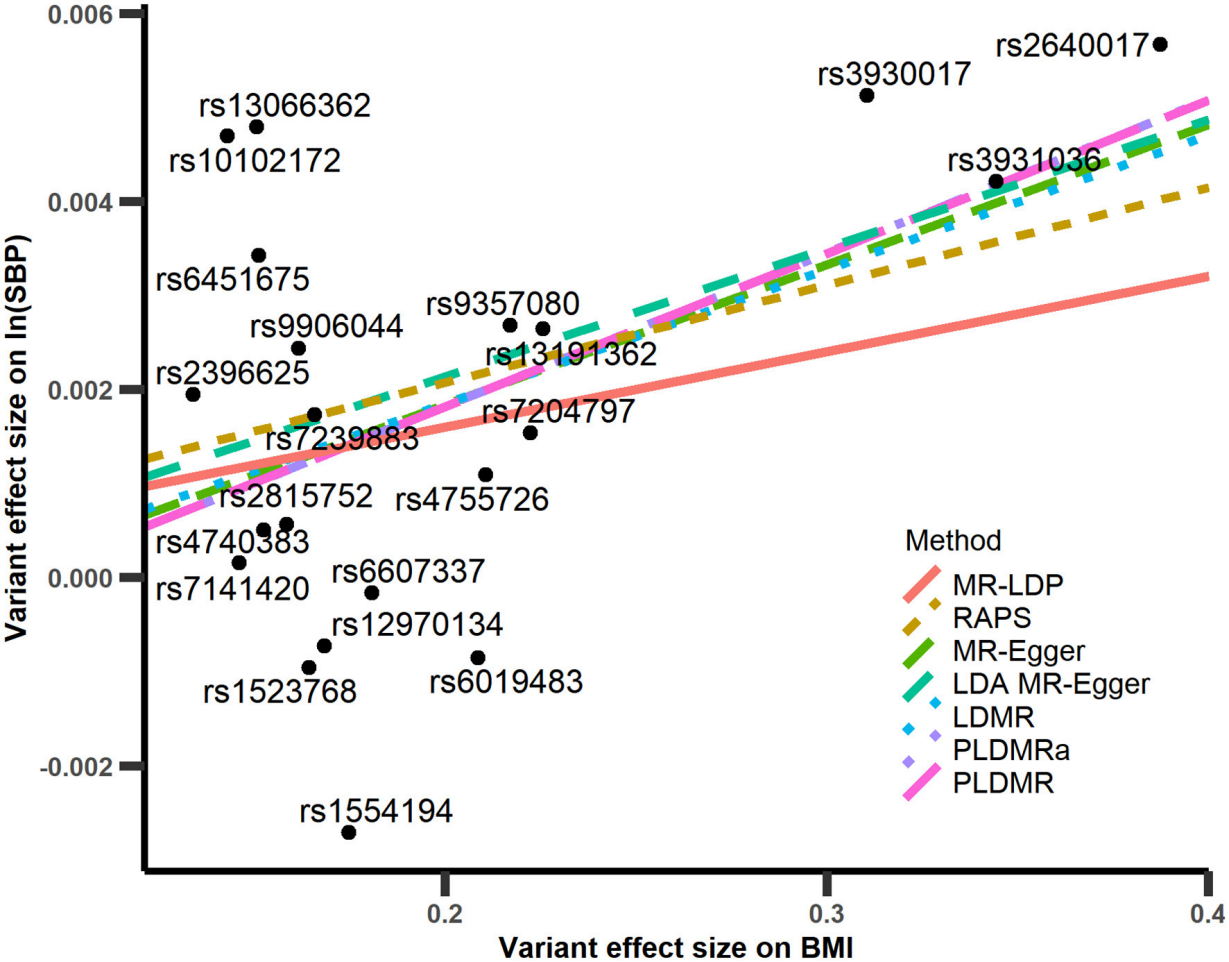
Scatter plot of $\hat{\Gamma }$ with respect to $\hat{\gamma }$ in the analyses of BMI-SBP. The red line is the regression line of MR-LDP method, the brown dashed line is the regression line of RAPS, the yellow-green dashed line is the regression line of MR-Egger method, the green dashed line is the regression line of LDA MR-Egger method, the blue short dashed line is the regression line of LDMR method, the purple short dashed line is the regression line of PLDMR_a_, and the magenta long dashed line is the regression line of PLDMR.

The results of $\hat{\Gamma }$ and $\hat{\gamma }$ of 21 SNPs are depicted in Figure 5, and the estimated causal effects, standard errors, and *p*-values are listed in Table 1. The estimate of *r*^2^ is about 0.015, which implies that pleiotropy may exist for those 21 SNPs. The point estimate of the causal effect is 0.0162 with the standard error 0.00677. The result of PLDMR_a_ is similar to that of PLDMR, with estimator 0.0163 with standard error 0.00666 for causal effect of BMI on SBP, while the MR-Egger and LDMR methods give point estimates of 0.0149 (with standard error 0.00985) and 0.0143 (with standard error 0.00911) for causal effect, respectively. Most importantly, PLDMR_a_ and PLDMR imply a significant causal effect of BMI on SBP with *p*-values 0.0244 and 0.0272, while MR-Egger and LDMR show no significance in the causal relationship of BMI and SBP (*p*-value = 0.130 and 0.133, respectively). In addition, we conduct MR-LDP, LDA MR-Egger and RAPS methods by randomly selecting 1,000 individuals from the whole dataset to estimate reference LD correlation matrix and splitting the remained 5,782 individuals into two halves to obtain summary statistics. The estimates of the causal effect given by MR-LDP and LDA MR-Egger are 0.00802 and 0.0136, respectively (with standard errors 0.00510 and 0.0109, respectively), which show no significance in the causal relationship between BMI and SBP (*p*-value = 0.116 and 0.228, respectively). RAPS estimates the causal effect as 0.0104 (with standard error 0.00362) and implies a significant causal relationship between BMI and SBP (*p*-value = 0.00419).

**Table 1:** Causal inference of BMI on SBP and GLU, respectively, in analyzing ARIC dataset.

	**SBP**			**GLU**		
**Method**	**β**	**Standard error**	***p*-value**	**β**	**Standard error**	***p*-value**
MR-LDP	0.0080	0.0051	0.1162	0.0055	0.0036	0.1349
RAPS	0.0104	0.0036	0.0042	0.0053	0.0025	0.0344
MR-Egger	0.0149	0.0098	0.1301	−0.0001	0.0066	0.9826
LDA MR-Egger	0.0136	0.0110	0.2280	−0.0012	0.0091	0.8919
LDMR	0.0143	0.0091	0.1330	−0.0019	0.0078	0.8108
PLDMR_a_	0.0163	0.0067	0.0244	−0.0007	0.0062	0.9146
PLDMR	0.0163	0.0067	0.0248	−0.0007	0.0062	0.9139

*The threshold of p-value for selecting SNPs is 5 × 10^−8^. The total number of SNPs is 21*.

Existing studies have already shown that there is a relationship between BMI and blood pressure or hypertension (Feng et al., 2012; Shihab et al., 2012; Hall et al., 2015). Recently, a population-based cohort study from UK Biobank including 120,000 individuals identified the association between BMI and hypertension, SBP and DBP, where Mendelian randomization was used to show significant positive association between BMI and SBP with *p*-value 2 × 10^−4^ (Lyall et al., 2017). In addition, a MR analysis is conducted by studying a total of 19,502 people from 36 study populations of European descents, confirming that BMI has causal relationship with SBP with *p*-value 6.7 × 10^−76^ (Fall et al., 2013). These results all support the conclusion inferred from our method. So when pleiotropy exists and can not be ignored, our method PLDMR is recommended.

### 3.4. Causal Inference of BMI on GLU

We also investigate the relationship between BMI and GLU (Supplementary Figure 11). The estimate of *r*^2^ is 0.000406, which means the pleiotropic effect is relatively small. Only RAPS shows a significant causal association between BMI and GLU ($\hat{\beta }$ = 0.00527 with standard error 0.00249 and *p*-value 0.0344). A large-scale MR study investigating a European population (34,538 people) concluded no significant association of BMI with glucose deterioration with *p*-value 0.787 (Wang et al., 2018). No statistical significance between BMI and fasting glucose was reported in another study (Xu et al., 2020) (*p*-value 0.546). The results of PLDMR are consistent with the findings in the literature.

## 4. Discussion

### 4.1. Relation Between PLDMR and Other Existing Methods

Many methods have been proposed to detect the invalid instrumental variables involved in Mendelian randomization analysis and then to correct the estimate of causal effect. For example, the Q test employs Cochran's Q statistic, which follows χ^2^ distribution with one degree of freedom, to detect outliers and exclude them out in further analysis of the summary level data (Bowden et al., 2018). They also proposed a scale factor ϕ, which is associated with the squared ratio $r^{2}=\sigma_{\alpha }^{2}/\sigma_{Y}^{2}$, to quantify the degree of heterogeneity in the *Q*-test (Bowden et al., 2018). Similar to the *Q*-test method, MR-PRESSO (Verbanck et al., 2018) first conducts a global test to detect the overall pleiotropic effect in a MR study, and then applies outlier test to rule out invalid genetic variants in follow-up study. Unlike the *Q*-test and MR-PRESSO methods, PLDMR contains all of the genetic variants associated with the exposure in a MR study and corrects the causal effect estimate with the mean and variance of pleiotropic effect.

Another strategy for adjusting the pleiotropy in MR studies is to additionally assume that the number of invalid genetic variants is less than half of the total number of variants, like the weighted median estimator and sisVIVE (Bowden et al., 2016; Kang et al., 2016). Adaptive lasso (Windmeijer et al., 2019) has been applied to select valid IVs and propose consistent estimate for causal effect by combining weighted median method with sisVIVE for individual level data. With these additional conditions on pleiotropic effect, it has been proved that α is estimable (Kang et al., 2016) and identification of the true set of invalid genetic variants is consistent (Windmeijer et al., 2019). However, when these conditions are not met (for example, the fraction of invalid genetic variants is >50%), these methods fail to give a proper estimate of causal effect.

TWMR (Porcu et al., 2019) is similar to multivariable MR (Burgess and Thompson, 2015), which takes multiple expression quantitative trait loci as exposures to control the pleiotropic effects mediated by expression loci to the trait. However, any other pleiotropic effects mediated by environmental factors such as diet and education can still be potential confounders which affect the performances of these two methods. Moreover, we have conducted simulation studies to verify the performance of TWMR. Because we only consider one exposure in this study, the TWMR is unable to identify the pleiotropic effects in this case and thus the results cannot meet expectations. Furthermore, GSMR (Zhu et al., 2018) can also be applied to two-sample summary-level data. It solves the pleiotropy and LD problems by excluding the SNPs which have pleiotropic effect and/or strong LD correlations between each other (Zhu et al., 2018). We also conduct simulation studies to compare our method and GSMR. As all the SNPs have pleiotropic effects and most of them are correlated with each other in our simulation study, the number of remaining SNPs after HEIDI test and LD pruning procedures may be <10, which would cause the instability warning in executing GSMR. In addition, it is observed from Figures 2A,C (Cheng et al., 2020) in the comparison of MR-LDP and GSMR that GSMR is not able to control type I error rate well when $h_{\alpha }^{2}$ is not zero, which is equivalent to the parameter setting of μ_α_ = 0 and $\sigma_{\alpha }^{2}>0$ in our simulation, thus we have excluded GSMR from the comparison.

PLDMR takes a similar strategy to RAPS (Zhao et al., 2020), but PLDMR also borrows the idea from MR-Egger. To be precise, RAPS only models the variance of pleiotropic effects to correct for causal effect, while PLDMR models both the mean and variance of pleiotropic effects. What is more, RAPS assumes the selected genetic variants are in linkage equilibrium but PLDMR allows the existence of LD in all IVs. Similar to RAPS, MR-LDP (Cheng et al., 2020) also models the variance of pleiotropic effects, which regards pleiotropic effects as latent variables and uses expectation-maximization (EM) algorithm to estimate the causal effect. LDA MR-Egger (Barfield et al., 2018) improves MR-Egger when LD exists among the selected SNPs. The estimator derived from LDA MR-Egger is actually quite similar to that of LDMR, despite of a little difference in weight matrices.

### 4.2. Limitations and Forecast of PLDMR

We have shown in Figure 1 that the small sample size *n* and high LD may cause type I error rate inflation, although very slight, for PLDMR method. This may mainly be caused by the relatively large variance of PLDMR estimator when the sample size *n* is small, since the term (***G***^*T*^***G***)^−1^ in the variance is associated with *n* and the diagonal elements of this matrix decrease at rate $\frac{1}{n}$. On the other hand, the slow growth of PLDMR's power under large variance of pleiotropic effect (Supplementary Figure 9) may be interpreted as “the strong pleiotropy in MR studies can dominate the power growth benefit from the increase in sample size.”

Furthermore, although we propose a measurement $r^{2}=\sigma_{\alpha }^{2}/\sigma_{Y}^{2}$ to describe the relative strength of pleiotropy, we have not developed a method to test for the potential pleiotropy in genetic variants. The test for pleiotropic effect is important as it adds the interpretability of MR analyses when PLDMR returns a different result from the traditional MR methods which do not take pleiotropy into account. MR-Egger models pleiotropy in the intercept term of the Egger's regression, thus the test for pleiotropy is equivalent to test whether the intercept in regression is zero (Bowden et al., 2015), while the *Q*-test in fact focuses on the regression residuals (Bowden et al., 2018). When testing pleiotropic effect with PLDMR, it is important to notice that we must test two parameters μ_α_ and *r*^2^ simultaneously, which is similar to the random-effects model in meta analysis (Han and Eskin, 2011) and may be conducted by the likelihood ratio test with a mixture of χ^2^ distributions.

PLDMR also has restrictions on the data involved. Because of the requirement of matrix ***G***^*T*^***G*** in calculating multiple regression coefficients $\hat{\Gamma },\hat{\gamma }$ and weight matrix $W^{\frac{1}{2}}$, individual data is needed for PLDMR method, whereas two-sample MR methods like MR-Egger (Bowden et al., 2015) only need summary level data and thus can be easily implemented using results from online database like GWAS Catalog. To extend the application of PLDMR in summary level data, similar to most MR methods which consider LD in summary level data analyses (Zhu et al., 2018; Porcu et al., 2019), we can approximately substitute the matrix ***G***^*T*^***G*** with the reference LD panels such as 1000Genomes or even ARIC dataset itself. This is work left for future study.

Ultimately, we conclude that although MR-Egger allows correction for LD, the type I error of testing the null hypothesis of *H*_0_ : β = 0 still inflates when directional pleiotropy and LD simultaneously exist between genetic variants, and LDA MR-Egger also fails to control type I error rate when there exists strong LD between genetic variants. PLDMR method controls type I error rate well and stays consistent with true value plug-in method PLDMR_t_, especially when MR-LDP and RAPS are unable to control type I error rates in the cases of directional pleiotropic effects. We further conclude that LDMR and PLDMR_a_ are effective approximation of PLDMR method when the variation of pleiotropy is small and the sample size is large, respectively.

## Data Availability Statement

The datasets ARIC for this study can be applied from the dbGaP Study https://www.ncbi.nlm.nih.gov/projects/gap/cgi-bin/study.cgi?study_id=phs000090.v4.p1. GWAS Catelog is available at https://www.ebi.ac.uk/gwas/. The R package MendelianRandomization is available at https://cran.r-project.org/web/packages/MendelianRandomization/index.html. The R package BB is available at https://cran.r-project.org/web/packages/BB/index.html. The R package MR.LDP is available at https://github.com/QingCheng0218/MR.LDP. The R package mr.raps is available at https://github.com/qingyuanzhao/mr.raps. The R code for LDA MR-Egger is available at https://rbarfield.github.io/Barfield_website/pages/Rcode.html.

## Ethics Statement

Written informed consent was obtained from the individual(s) for the publication of any potentially identifiable images or data included in this article.

## Author Contributions

YW and TL developed the proposed method, designed the simulation study, and wrote the initial draft of manuscript. Y-QH contributed to the development of the method and to drafting the manuscript. LF preprocessed the data and guided data analysis. SY reviewed and approved the final manuscript. All authors contributed to the article and approved the submitted version.

## Conflict of Interest

The authors declare that the research was conducted in the absence of any commercial or financial relationships that could be construed as a potential conflict of interest.

## Supplementary Material

The source code for generating the simulation results and PLDMR method can be found in https://github.com/YQHuFD/PLDMR.
