# Supplementary material for: A Novel Method for Mendelian Randomization Analyses With Pleiotropy and Linkage Disequilibrium in Genetic Variants From Individual Data
Source: Front Genet. 2021 Jul 12;12:634394. doi: 10.3389/fgene.2021.634394 (PMC8312241; doi:10.3389/fgene.2021.634394)
Supplement: Supplementary file 1 [file Data_Sheet_1.PDF]

Supplementary Material

1 SUPPLEMENTARY TABLES AND FIGURES

1.1 Figures

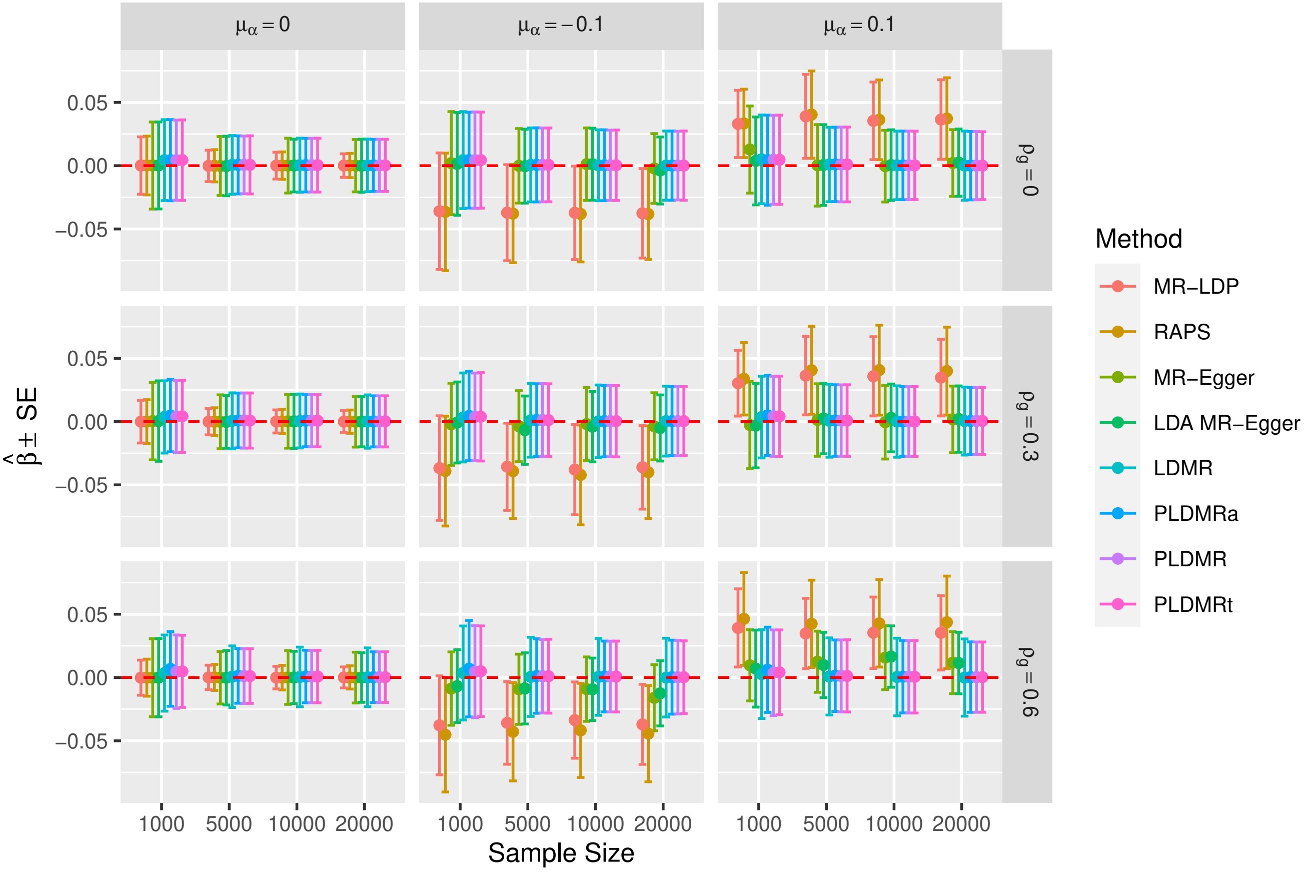

Figure S1: Plot of the performances of all eight estimating methods when $\beta = 0$. Sample size $n = 1\ 000, 5\ 000, 10\ 000, 20\ 000$, the number of genetic variants $m = 25$, and $\sigma_\alpha = 0.1$. $\mu_\alpha = 0, -0.1, 0.1$ represents the mean of pleiotropic effect and $\rho_g = 0, 0.3, 0.6$ stands for the relative strength of LD between the genetic variants. The solid circles are the mean values of estimators, the upper and lower bars are the means plus and minus one standard error in 10 000 replications. The red dashed line indicates the true value of β.

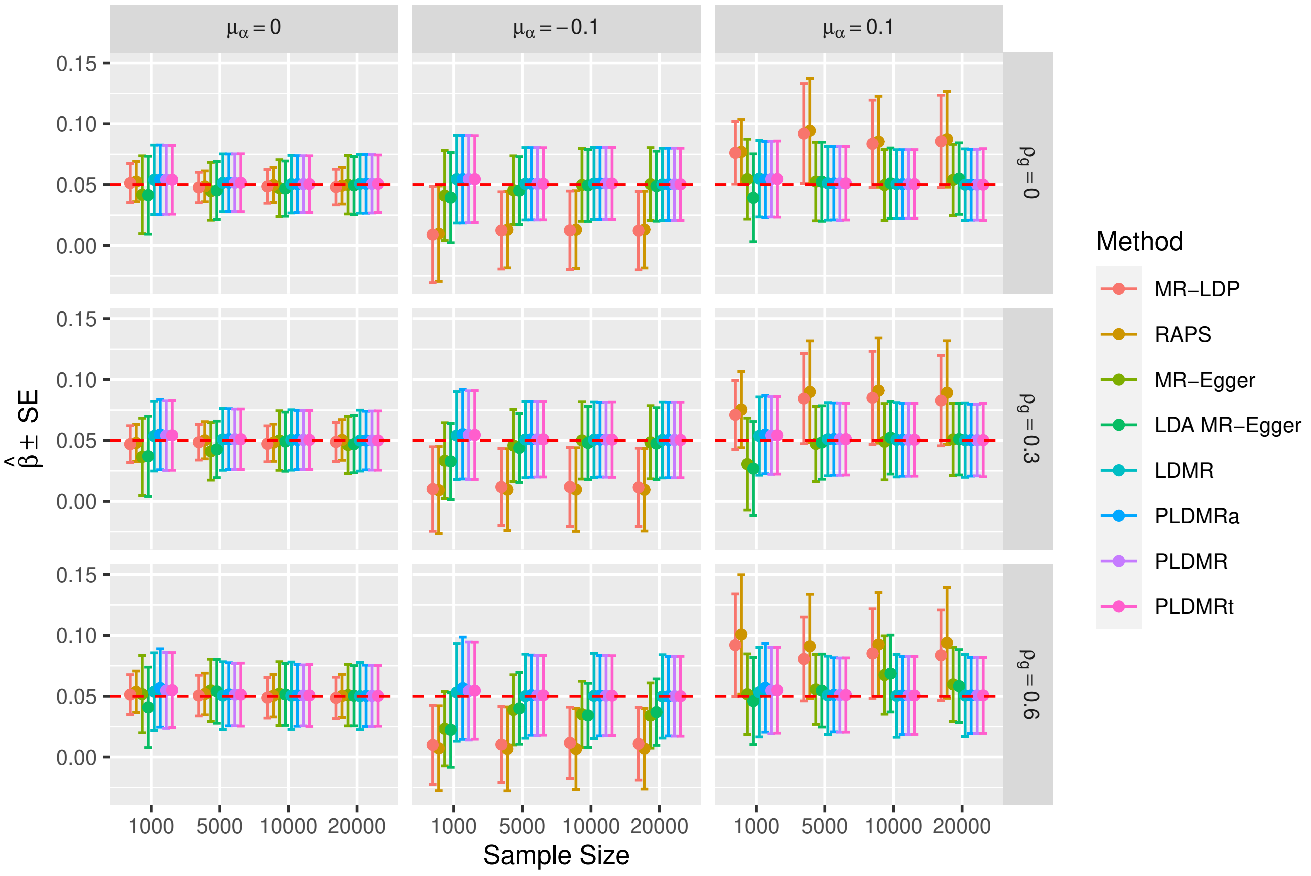

Figure S2: Plot of the performances of all eight estimating methods when $\beta = 0.05$. Sample size $n = 1\,000, 5\,000, 10\,000, 20\,000$, the number of genetic variants $m = 25$, and $\sigma_\alpha = 0.1$. $\mu_\alpha = 0, -0.1, 0.1$ represents the mean of pleiotropic effect and $\rho_g = 0, 0.3, 0.6$ stands for the relative strength of LD between the genetic variants. The solid circles are the mean values of estimators, the upper and lower bars are the means plus and minus one standard error in 10 000 replications. The red dashed line indicates the true value of β.

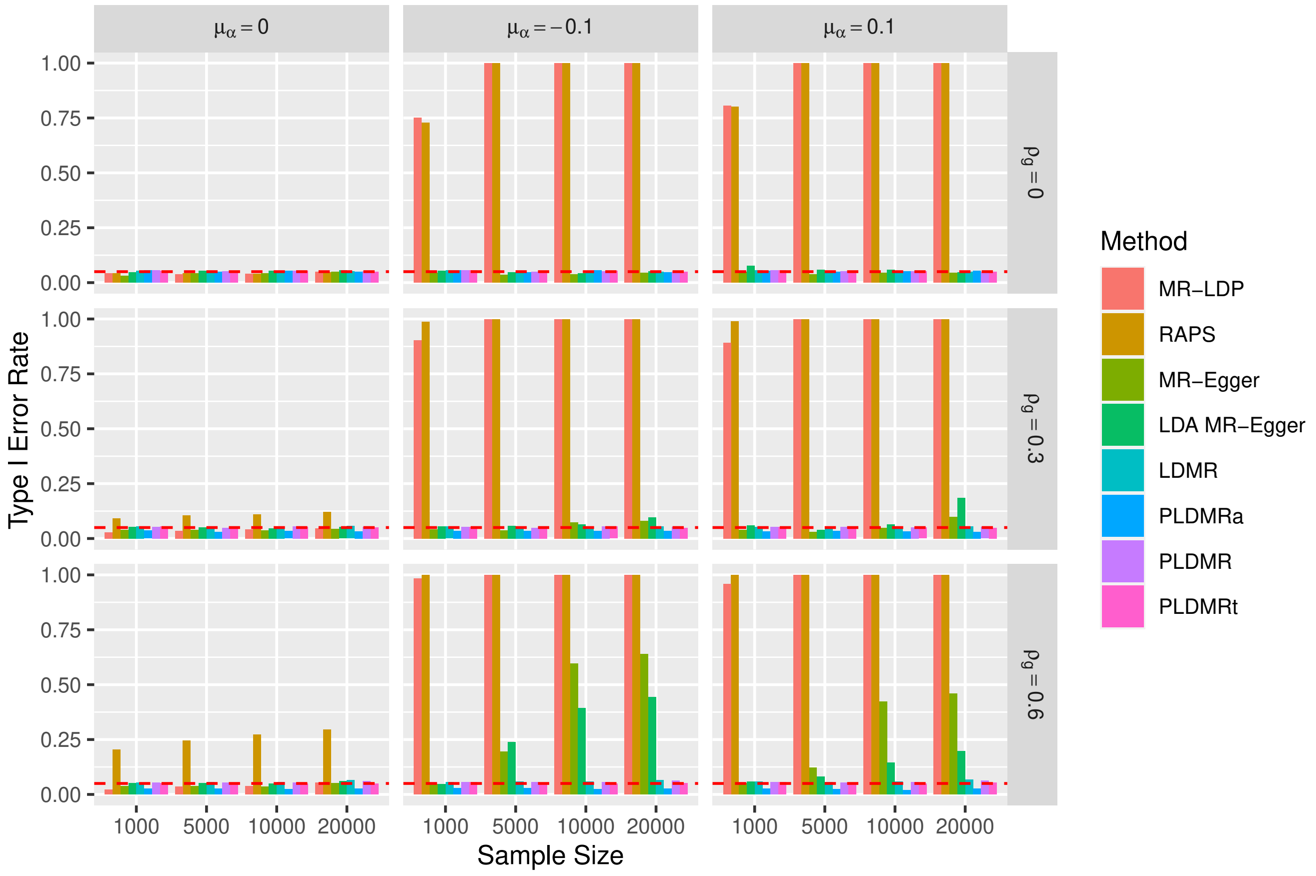

Figure S3: Bar plot of the type I error rates of all methods under the null hypothesis of $H_0 : \beta = 0$. Sample size $n = 1\ 000, 5\ 000, 10\ 000, 20\ 000$, the number of genetic variants $m = 25$, and $\sigma_\alpha = 0.01$. $\mu_\alpha = 0, -0.1, 0.1$ represents the mean of pleiotropic effect and $\rho_g = 0, 0.3, 0.6$ stands for the relative strength of LD between the genetic variants. The red dashed horizontal line indicates the nominal significance of 0.05.

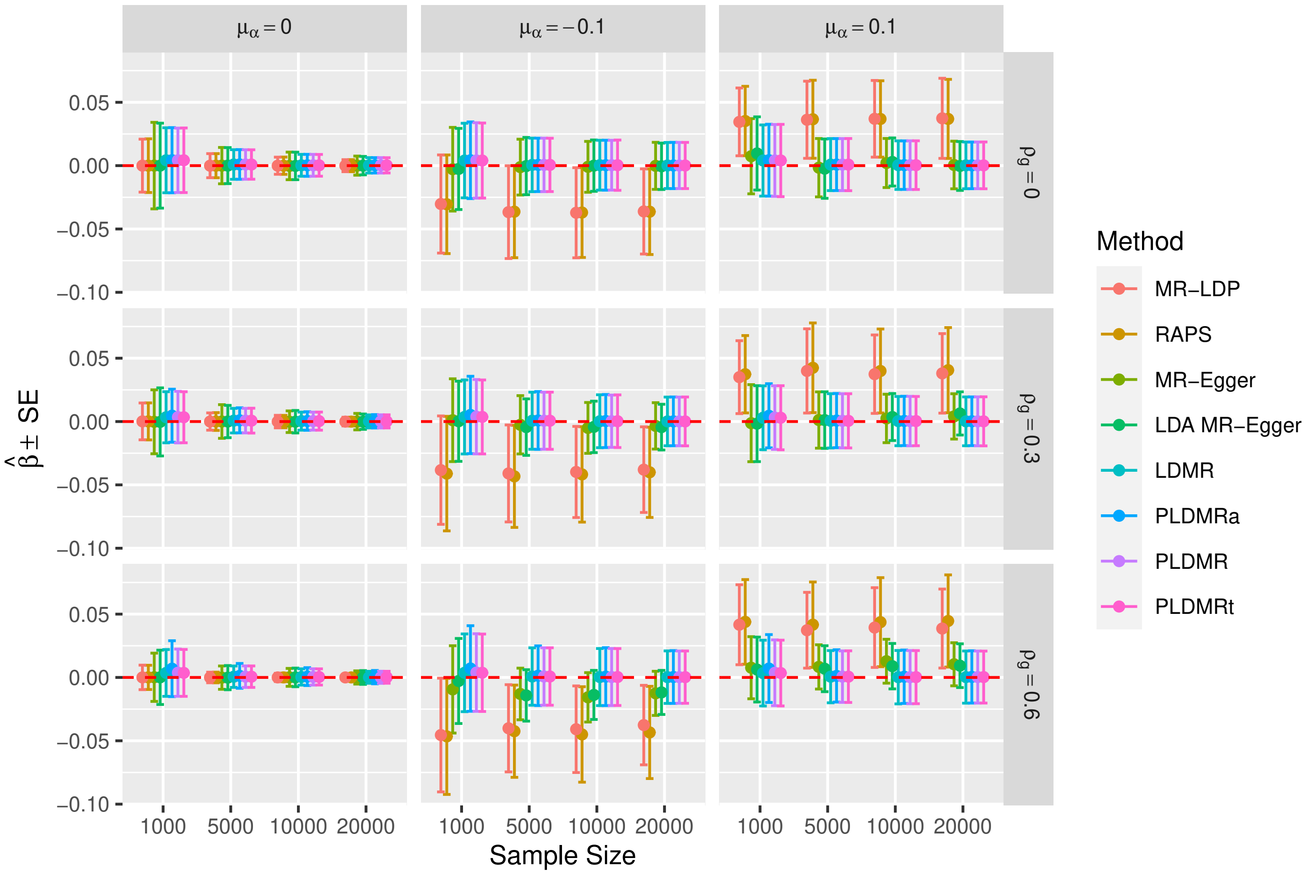

Figure S4: Plot of the performances of all eight estimating methods when $\beta = 0$. Sample size $n = 1\ 000, 5\ 000, 10\ 000, 20\ 000$, the number of genetic variants $m = 25$, and $\sigma_\alpha = 0.01$. $\mu_\alpha = 0, -0.1, 0.1$ represents the mean of pleiotropic effect and $\rho_g = 0, 0.3, 0.6$ stands for the relative strength of LD between the genetic variants. The solid circles are the mean values of estimators, the upper and lower bars are the means plus and minus one standard error in 10 000 replications. The red dashed line indicates the true value of β.

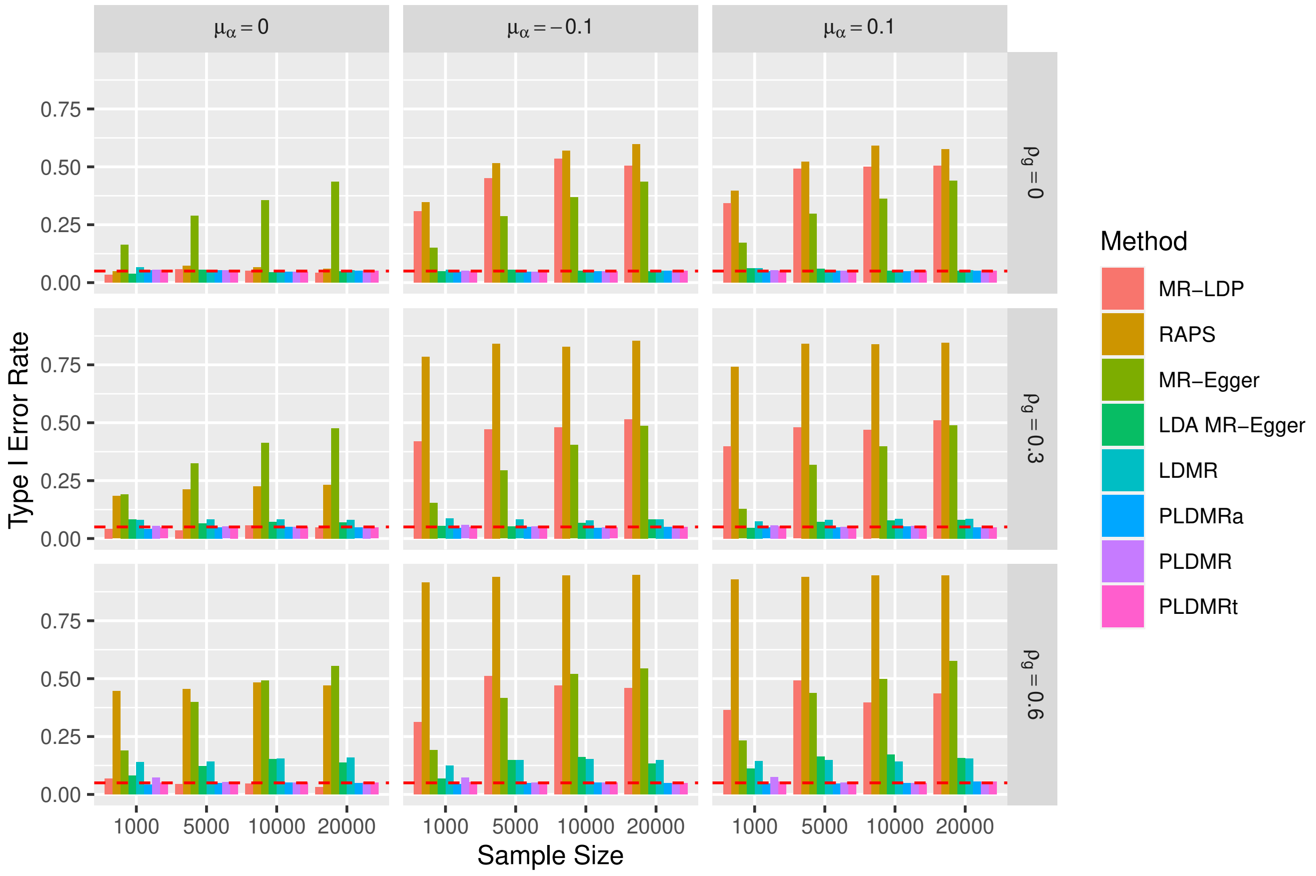

Figure S5: Bar plot of the type I error rates of all methods under the null hypothesis of $H_0 : \beta = 0$. Sample size $n = 1\ 000, 5\ 000, 10\ 000, 20\ 000$, the number of genetic variants $m = 25$, and $\sigma_\alpha = 0.2$. $\mu_\alpha = 0, -0.1, 0.1$ represents the mean of pleiotropic effect and $\rho_g = 0, 0.3, 0.6$ stands for the relative strength of LD between the genetic variants. The red dashed horizontal line indicates the nominal significance of 0.05.

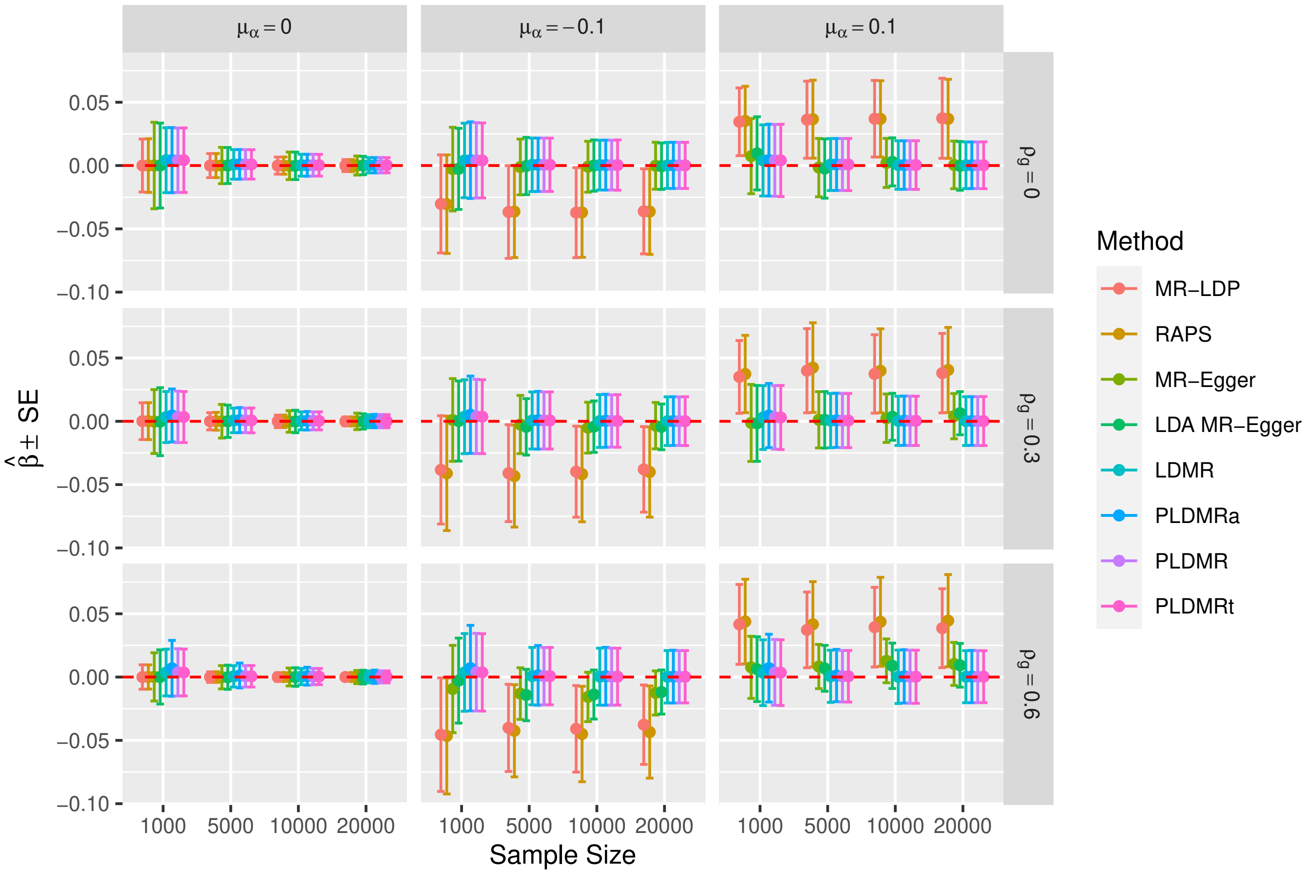

Figure S6: Plot of the performances of all eight estimating methods when $\beta = 0$. Sample size $n = 1\ 000, 5\ 000, 10\ 000, 20\ 000$, the number of genetic variants $m = 25$, and $\sigma_\alpha = 0.01$. $\mu_\alpha = 0, -0.1, 0.1$ represents the mean of pleiotropic effect and $\rho_g = 0, 0.3, 0.6$ stands for the relative strength of LD between the genetic variants. The solid circles are the mean values of estimators, the upper and lower bars are the means plus and minus one standard error in 10 000 replications. The red dashed line indicates the true value of β.

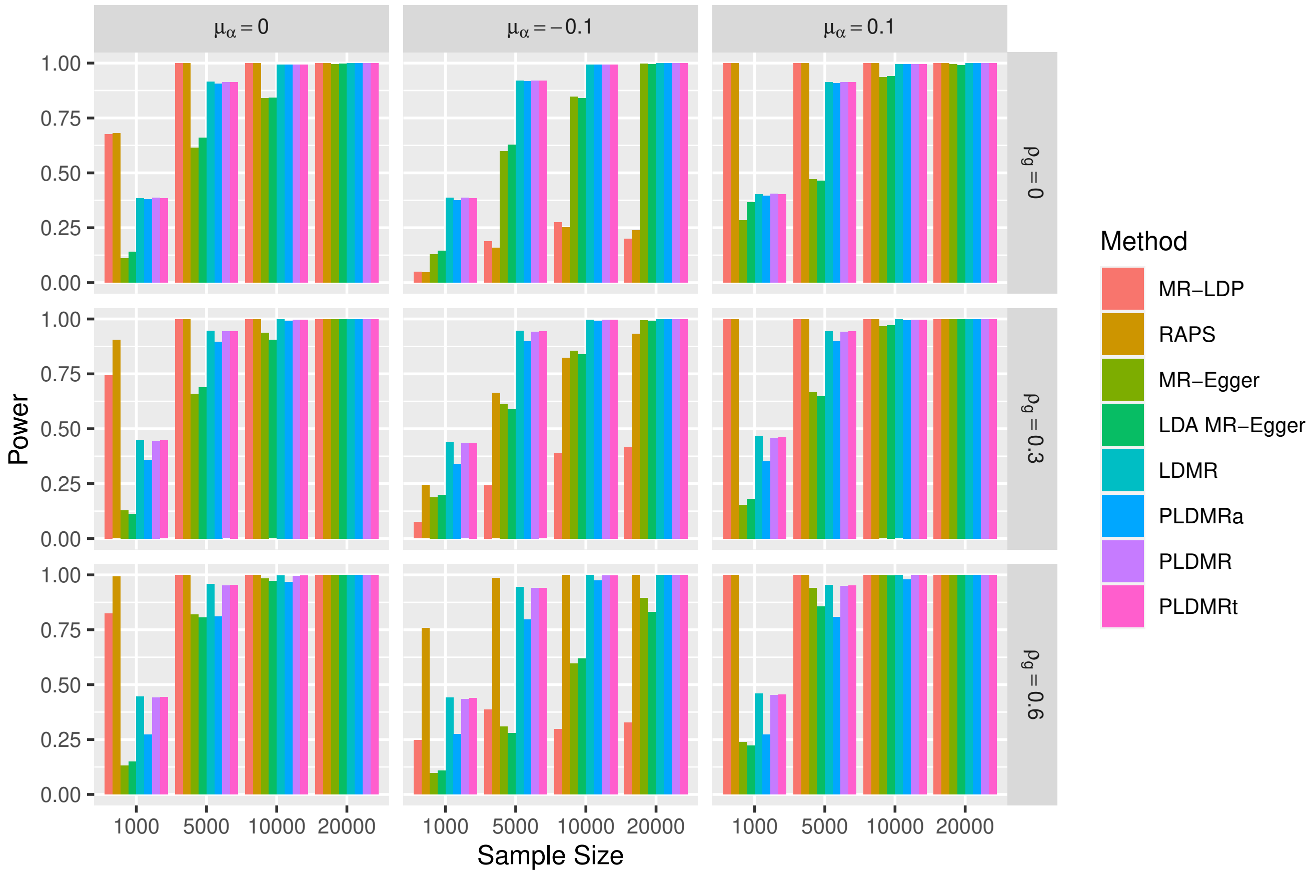

Figure S7: Bar plot of the powers of all methods under the alternative hypothesis of $H_1 : \beta = 0.03$. Sample size $n = 1\ 000, 5\ 000, 10\ 000, 20\ 000$, the number of genetic variants $m = 25$, and $\sigma_\alpha = 0.01$. $\mu_\alpha = 0, -0.1, 0.1$ represents the mean of pleiotropic effect and $\rho_g = 0, 0.3, 0.6$ stands for the relative strength of LD between the genetic variants.

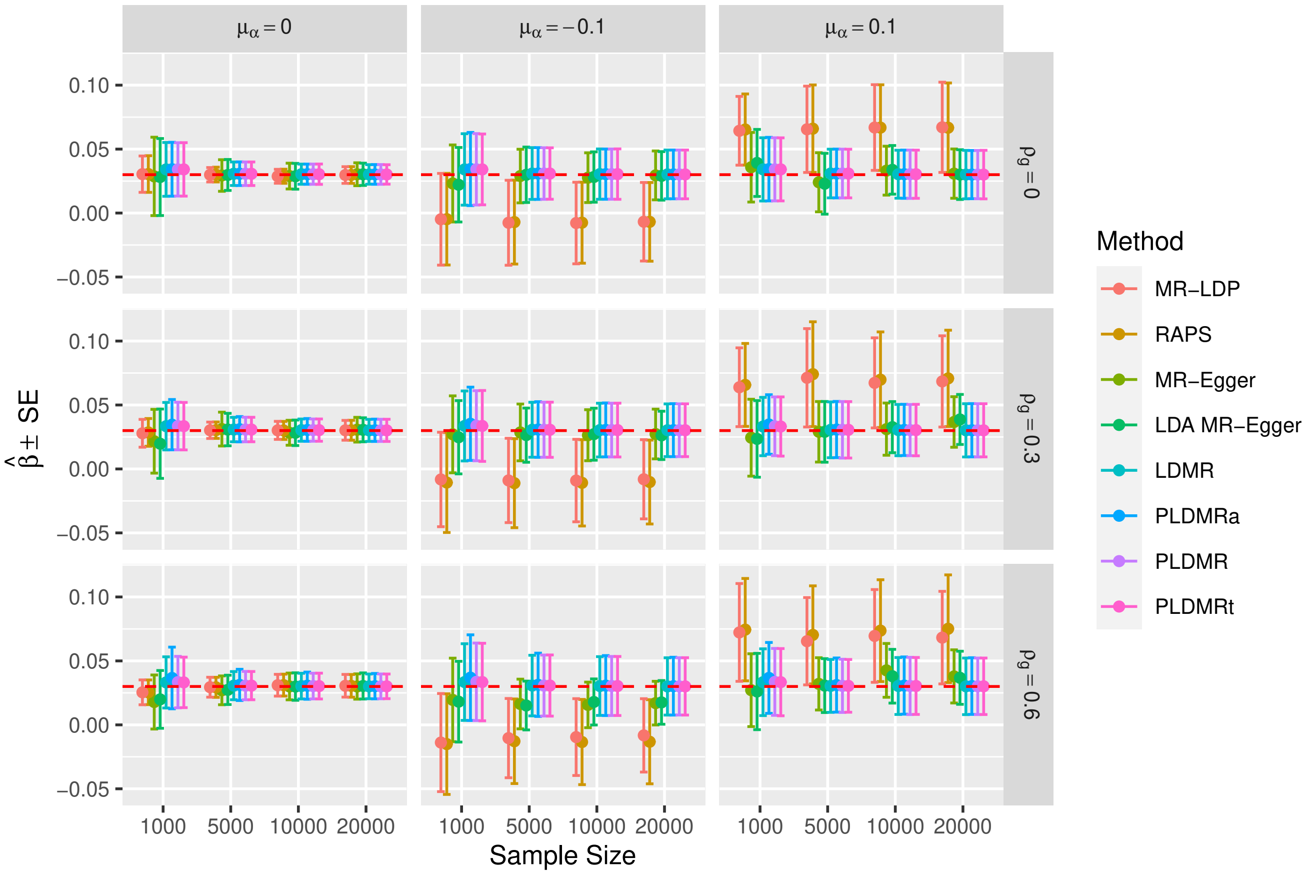

Figure S8: Plot of the performances of all eight estimating methods when $\beta = 0.03$. Sample size $n = 1\,000, 5\,000, 10\,000, 20\,000$, the number of genetic variants $m = 25$, and $\sigma_\alpha = 0.01$. $\mu_\alpha = 0, -0.1, 0.1$ represents the mean of pleiotropic effect and $\rho_g = 0, 0.3, 0.6$ stands for the relative strength of LD between the genetic variants. The solid circles are the mean values of estimators, the upper and lower bars are the means plus and minus one standard error in 10 000 replications. The red dashed line indicates the true value of β.

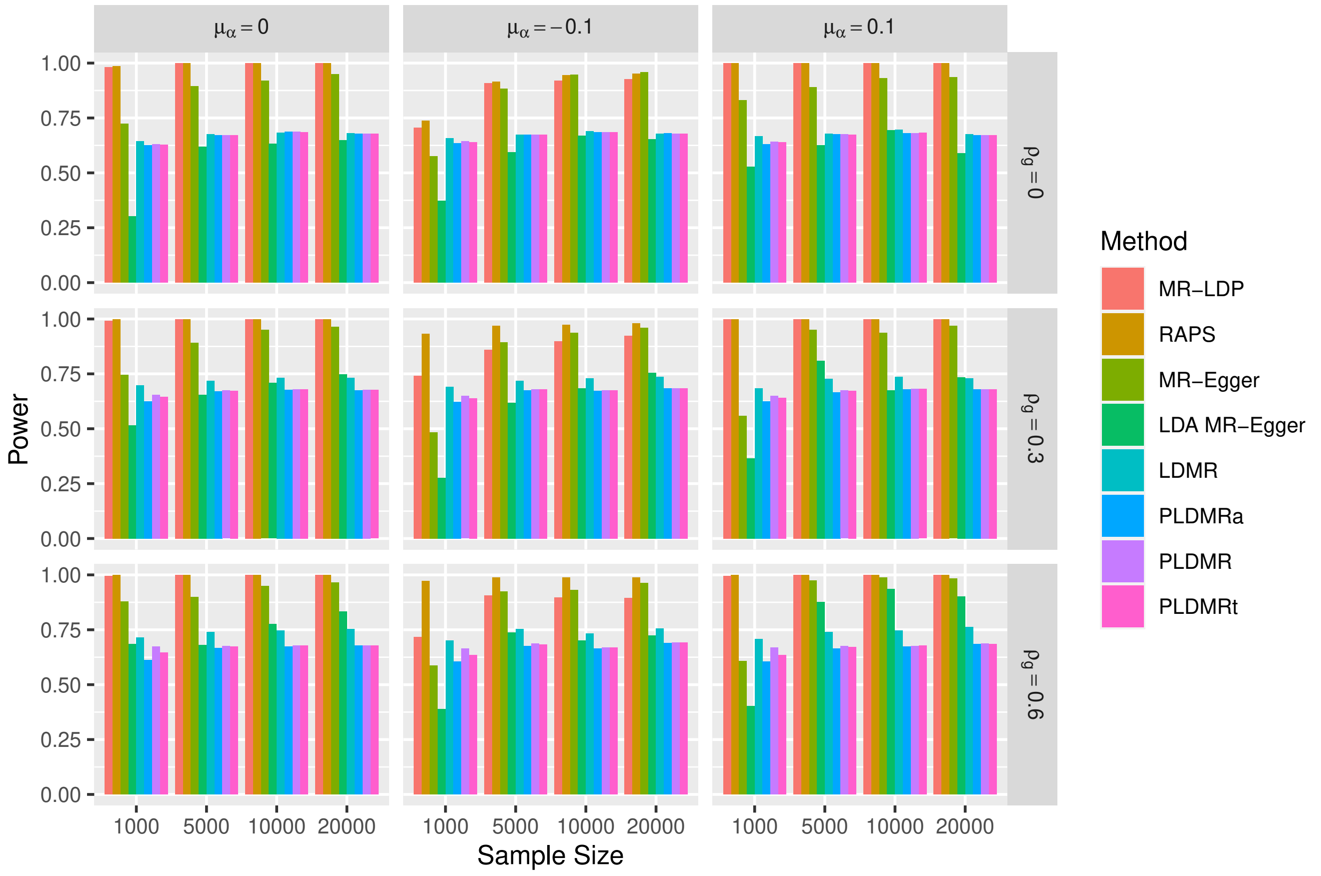

Figure S9: Bar plot of the powers of all methods under the alternative hypothesis of $H_1 : \beta = 0.1$. Sample size $n = 1\ 000, 5\ 000, 10\ 000, 20\ 000$, the number of genetic variants $m = 25$, and $\sigma_\alpha = 0.2$. $\mu_\alpha = 0, -0.1, 0.1$ represents the mean of pleiotropic effect and $\rho_g = 0, 0.3, 0.6$ stands for the relative strength of LD between the genetic variants.

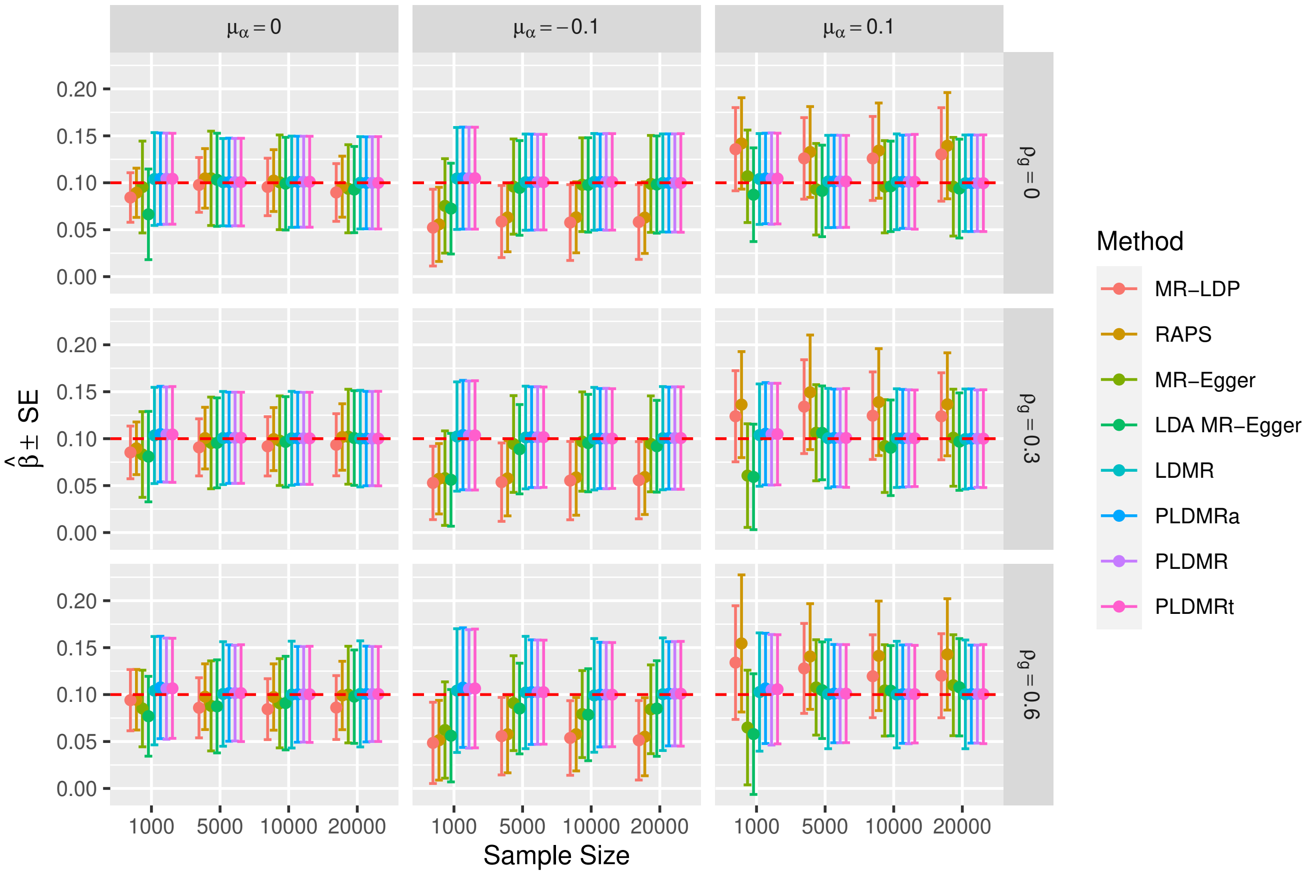

Figure S10: Plot of the performances of all eight estimating methods when $\beta = 0.1$. Sample size $n = 1\,000, 5\,000, 10\,000, 20\,000$, the number of genetic variants $m = 25$, and $\sigma_\alpha = 0.2$. $\mu_\alpha = 0, -0.1, 0.1$ represents the mean of pleiotropic effect and $\rho_g = 0, 0.3, 0.6$ stands for the relative strength of LD between the genetic variants. The solid circles are the mean values of estimators, the upper and lower bars are the means plus and minus one standard error in 10 000 replications. The red dashed line indicates the true value of β.

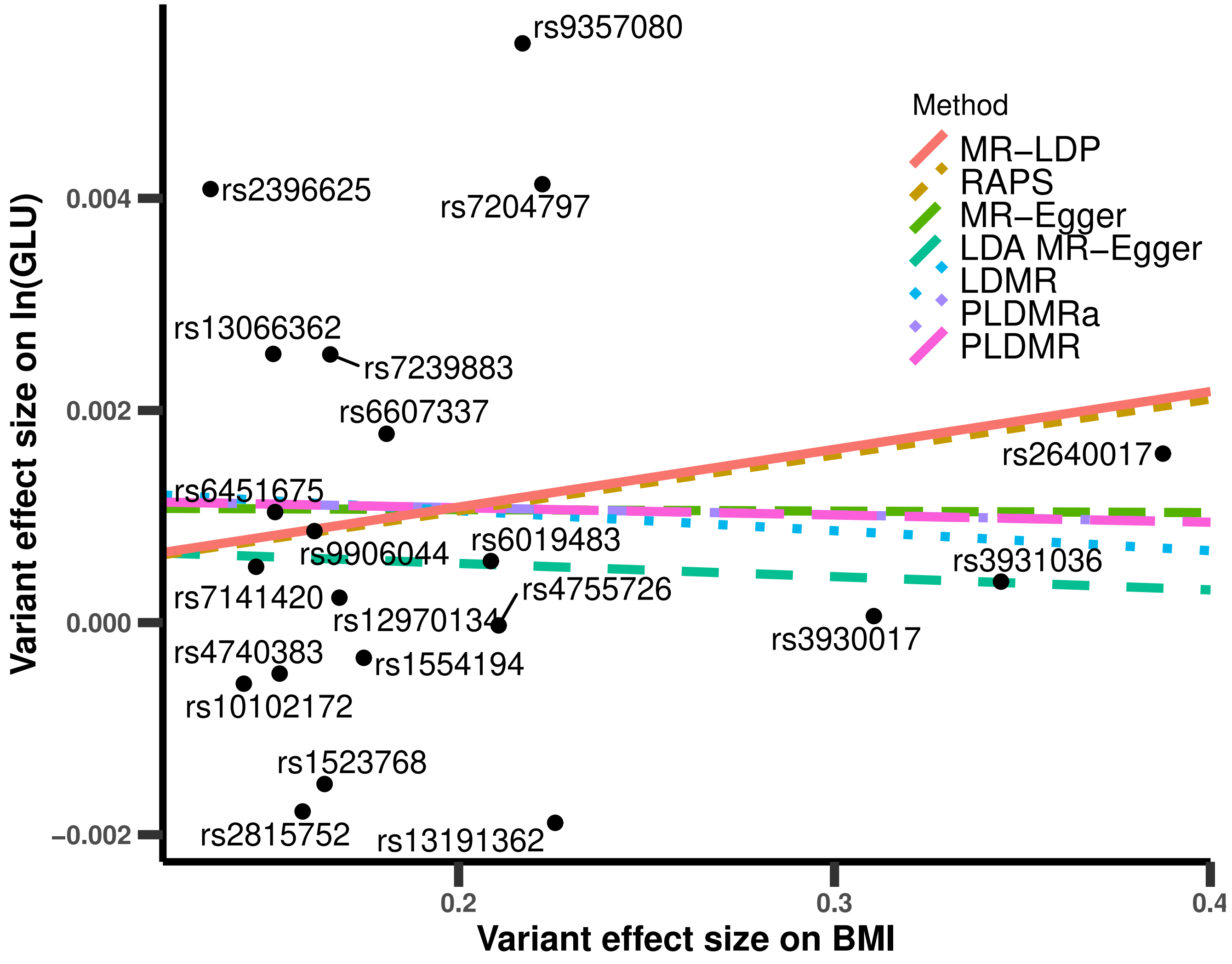

Figure S11: Scatter plot of $\hat{\Gamma}$ with respect to $\hat{\gamma}$ in the analyses of BMI-GLU. The red line is the regression line of MR-LDP method, the brown dashed line is the regression line of RAPS, the yellow-green dashed line is the regression line of MR-Egger method, the green dashed line is the regression line of LDA MR-Egger method, the blue short dashed line is the regression line of LDMR method, the purple short dashed line is the regression line of $PLDMR_a$ and the magenta long dashed line is the regression line of PLDMR.

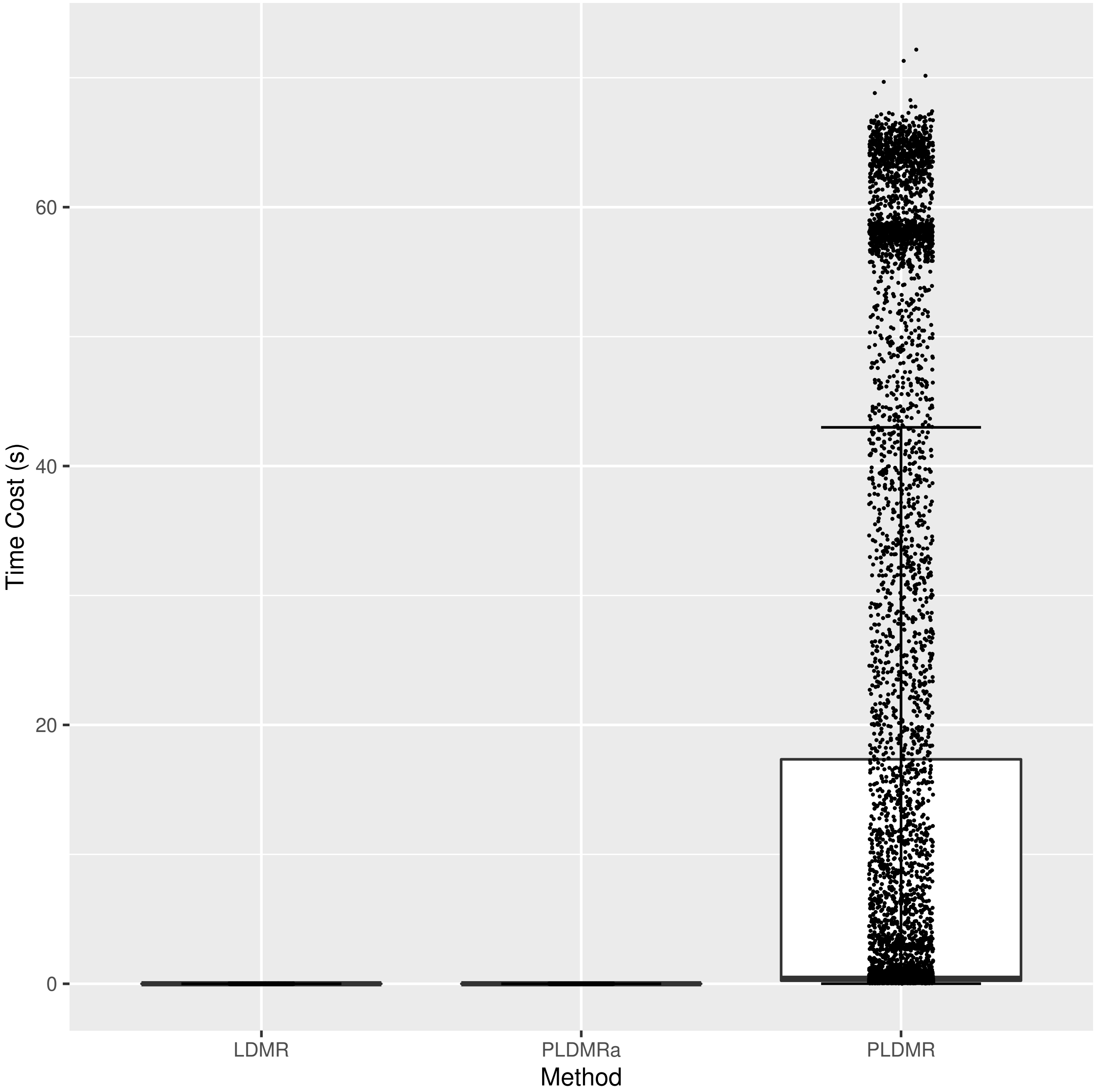

Figure S12: Boxplots of time costs of LDMR, PLDMR and $\mathrm{PLDMR_a}$ when $\beta = 0$. Sample size is $n = 5000$, the number of genetic variants is $m = 25$ and $\sigma_\alpha = 0.1$. $\mu_\alpha = 0.1$ and $\rho_g = 0.6$. The points are time spent by methods at each replication.

1.2 Tables

SNP	Chr	Position(bp)	Genes(nearest)	Alleles(effect)	Alleles(other)	β	SE	p-value	EAF
rs2815752	1	72346757	NEGR1	A	G	0.150	0.067	0.026	0.635
rs2640017	3	141616279	RASA2	G	A	0.350	0.134	0.009	0.079
rs1554194	3	8096354	AC018832.1	C	G	0.155	0.066	0.019	0.493
rs13066362	3	158291688	RSRC1	C,G,T	A	0.155	0.067	0.020	0.398
rs1523768	3	77617893	ROBO2	A	G	0.172	0.070	0.014	0.708
rs6451675	5	43110753	ZNF131	G,T	C	0.156	0.072	0.029	0.715
rs9357080	6	29355089	OR5V1	G	A	0.213	0.106	0.045	0.096
rs13191362	6	162612318	PARK2	G	A	0.226	0.101	0.025	0.093
rs3930017	7	77091265	FAM185BP	G,T	A	0.290	0.066	0.000	0.599
rs2396625	7	113388579	GPR85	A	T	0.145	0.067	0.031	0.421
rs10102172	8	76326157	ZFHX4	C,G	A	0.143	0.066	0.030	0.548
rs4740383	9	130908179	FIBCD1	A,C	G	0.150	0.067	0.025	0.447
rs4755726	11	43620580	HSD17B12	A,C,G	T	0.212	0.072	0.003	0.694
rs7141420	14	79433111	NRXN3	G,T	C	0.139	0.066	0.035	0.540
rs7204797	16	29956694	TMEM219	T	C	0.219	0.066	0.001	0.431
rs3931036	16	70514394	COG4	A,C	G	0.329	0.132	0.013	0.942
rs6607337	17	36700930	DHRS11	A,T	C	0.174	0.072	0.016	0.307
rs9906044	17	37251740	ACACA	T	A	0.182	0.071	0.010	0.344
rs7239883	18	42567706	LINC00907	A,T	G	0.159	0.068	0.020	0.612
rs12970134	18	60217517	MC4R	A	G	0.167	0.075	0.027	0.259
rs6019483	20	48879119	LOC105372648	A	T	0.190	0.089	0.033	0.155

Table S1. The information of SNPs involved in this study. The annotations are from GRCh38 human genome build. EAF means effective allele frequency.

Method	SBP			GLU		
	β	Standard Error	p-value	β	Standard Error	p-value
MR-LDP	0.0124	0.0058	0.0339	0.0031	0.0040	0.4328
RAPS	0.0104	0.0032	0.0013	0.0053	0.0022	0.0142
MR-Egger	0.0158	0.0095	0.0971	0.0043	0.0064	0.4978
LDA MR-Egger	0.0120	0.0109	0.2783	0.0039	0.0094	0.6785
LDMR	0.0160	0.0082	0.0605	0.0042	0.0074	0.5734
PLDMR_a	0.0202	0.0061	0.0024	0.0053	0.0056	0.3499
PLDMR	0.0202	0.0061	0.0025	0.0053	0.0056	0.3516

Table S2. Causal inference of BMI on SBP and GLU, respectively, in analysing ARIC dataset. The threshold of p-value for selecting SNPs is 1×10^{-4}. The total number of SNPs is 29.
